# Assessment of individual and conspecific reproductive success as determinants of breeding dispersal of female tree swallows: A capture–recapture approach

**DOI:** 10.1002/ece3.3241

**Published:** 2017-08-09

**Authors:** Paméla Lagrange, Olivier Gimenez, Blandine Doligez, Roger Pradel, Dany Garant, Fanie Pelletier, Marc Bélisle

**Affiliations:** ^1^ Département de Biologie Université de Sherbrooke Sherbrooke QC Canada; ^2^ CEFE UMR 5175 CNRS ‐ Université de Montpellier Université Paul‐Valéry Montpellier ‐ EPHE Montpellier Cedex 5 France; ^3^ Laboratoire de Biométrie et Biologie Evolutive ‐ CNRS UMR 5558 Université de Lyon 1 Villeurbanne France

**Keywords:** capture–recapture data, dispersal, multievent model, reproductive success, social information, tree swallow

## Abstract

Breeding dispersal is a key process of population structure and dynamics and is often triggered by an individual's breeding failure. In both colonial and territorial birds, reproductive success of conspecifics (RSc) can also lead individuals to change breeding sites after a failure on a site. Yet, few studies have simultaneously investigated the independent contribution of individual reproductive success (RSi) and of RSc on dispersal decision. Here, we develop a modeling framework to disentangle the effects of RSi and RSc on demographic parameters, while accounting for imperfect individual detection and other confounding factors such as age or dispersal behavior in the previous year. Using a 10‐year capture–recapture dataset composed of 1,595 banded tree swallows, we assessed the effects of nonmanipulated RSi and RSc on female breeding dispersal in this semicolonial passerine. Dispersal was strongly driven by RSi, but not by RSc. Unsuccessful females were 9.5–2.5 times more likely to disperse than successful ones, depending if they had dispersed or not in the previous year, respectively. Unsuccessful females were also three times less likely to be detected than successful ones. Contrary to theoretical and empirical studies, RSc did not drive the decision to disperse but influenced the selection of the following breeding site once dispersal had been initiated. Because detection of individuals was driven by RSi, which was positively correlated to RSc, assuming a perfect detection as in previous studies may have lead us to conclude that RSc affected dispersal patterns, yet our approach corrected for this bias. Overall, our results suggest that the value and use of RSc as public information to guide dispersal decisions are likely dictated by multiple ecological determinants, such as landscape structure and extent, if this cue is indeed used.

## 1 INTRODUCTION

Breeding dispersal, the movement between subsequent breeding sites, plays a key role in population structure and dynamics as well as evolution (Clobert, Danchin, Nichols, & Dhondt, 2001). This behavior is inherent to habitat selection, which can strongly determine the survival and reproductive success of individuals (Bowler & Benton, 2005). Such habitat choice implies a varying quality among breeding sites and that individuals can perceive and collect reliable information about habitat quality (Doligez, Pärt, & Danchin, 2004; Switzer, 1993). Information about the quality of sites can be obtained from environmental attributes, such as food availability (Ward, 2005) or predator density (Clobert et al., 2001; Ward, 2005), as well as from social factors, such as density (Betts, Hadley, Rodenhouse, & Nocera, 2008; Stamps, 1988), body condition, or reproductive success of conspecifics (RSc) (Brown, Brown, & Danchin, 2000; Dall, Giraldeau, Olsson, McNamara, & Stephens, 2005; Wagner & Danchin, 2010). To be adaptive, site choice also implies that breeding site quality is predictable between the time where information was collected and used (Doligez, Cadet, Danchin, & Boulinier, 2003; Switzer, 1993). Information from several cues must be collected through some form of prospecting during the previous breeding occasion or just before the onset of reproduction to decide whether or not to disperse (Clobert et al., 2001; Doligez, Pärt, & Danchin, 2004; Kivelä et al., 2014). Prospecting is indeed particularly well developed in nonbreeders or unsuccessful breeders, which tend to prospect potential future breeding sites more than successful breeders (Boulinier & Danchin, 1997; Boulinier, McCoy, Yoccoz, Gasparini, & Tveraa, 2008; Dittmann, Zinsmeister, & Becker, 2005; Ponchon et al., 2013; Ward, 2005).

Individuals can select breeding sites based on their own experience, such as their individual reproductive success (RSi) (i.e., personal information; Switzer, 1997; Danchin & Cam, 2002). They can also rely on inadvertent social information that consists of cues about the performance of others, such as the RSc (i.e., public information; Boulinier & Danchin, 1997; Danchin, Giraldeau, Valone, & Wagner, 2004). In birds, the number and condition of offspring, typically fledglings, are likely the most important cues because they represent the best proxy of the reproductive success obtained by individuals at a given site (Danchin, Heg, & Doligez, 2001). Many empirical studies have shown the importance of RSi on the decision to disperse or to remain in the same site for successive breeding attempts: Individuals with a higher RSi tend to be more philopatric (Greenwood & Harvey, 1982; Hoover, 2003; Johnson & Gaines, 1990; Switzer, 1997). Like RSi, RSc can drive dispersal. Indeed, in some colonial seabirds and territorial passerines and in raptors, individuals were more likely to disperse with a decreasing RSc (Danchin, Boulinier, & Massot, 1998; Doligez, Danchin, & Clobert, 2002; Parejo, White, Clobert, Dreiss, & Danchin, 2007; Serrano, Tella, Forero, & Donazar, 2001). Birds were also more likely to settle on sites that presented a high RSc in the previous year than on sites with a lower RSc (Calabuig, Ortego, Aparicio, & Cordero, 2008; Danchin et al., 1998; Doligez, Pärt, Danchin, Clobert, & Gustafsson, 2004; Ward, 2005). However, only a few empirical studies have jointly assessed the independent effect of RSi and RSc on dispersal decisions (Boulinier & Danchin, 1997; Danchin et al., 1998). The most comprehensive assessment was conducted by Danchin et al. (1998) on black‐legged kittiwakes, *Rissa tridactyla*, a colonial seabird. This study showed that individual breeding performance did not affect the probability of changing sites for birds breeding on cliffs showing a high RSc, but that an individual's breeding failure increased the probability to disperse when breeding on cliffs showing a poor RSc. Because the recapture probability was close to 1 between breeders and nonbreeders in the above study system (Cam & Monnat, 2000), the conclusions reached by Danchin et al. (1998) using logistic regressions, while assuming a perfect detection of individuals between years, are likely to hold. Nevertheless, none of the other studies that addressed the role of RSc on dispersal decisions have considered the potential biases on dispersal probability estimates that may result from an imperfect detection of marked individuals (e.g., overestimation of dispersal probabilities due to lower detection rates in certain habitats; Gimenez et al., 2008).

In this study, we assess the independent contribution of RSi and RSc on the breeding dispersal decision of female tree swallows, *Tachycineta bicolor*. Like most studies that addressed the role of RSi on dispersal in birds, a previous analysis of breeding dispersal of tree swallows found that females were more likely to disperse (28% vs. 5%) when they failed to fledge at least one young (Winkler et al., 2004). Yet, as most other studies of bird dispersal, that analysis assumed perfect detection and is thus subject to provide biased dispersal probability estimates. Our first objective was to estimate the effect of RSi on the probability to disperse between consecutive breeding events while correcting for bias caused by imperfect detection. Moreover, the effect of RSi on dispersal decisions may have been affected by that of RSc, which has never been investigated in tree swallows despite its semicolonial breeding habits. Thus, our second objective was to include the potential effect of RSc on the probability to disperse. To tackle these objectives, we adapted a multievent capture–recapture model (Lagrange, Pradel, Bélisle, & Gimenez, 2014) to quantify the independent effects of RSi and RSc on the decision to disperse in female tree swallows while accounting for imperfect detection. We therefore aimed to test the prediction that the likelihood of dispersal increases as RSi decreases, especially at low RSc, and thus assess the potential of RSc to override the effect of RSi on dispersal decisions as observed in Danchin et al. (1998).

## 2 MATERIALS AND METHODS

### 2.1 Model species

The tree swallow is a passerine that feeds on flying insects on the wing and which form loose colonies during the breeding season (Dunn & Hannon, 1991; Winkler et al., 2011). This long‐distance migrant breeds over much of northern North America up to its northern tree line and winters mostly in southern USA and Mexico (Winkler et al., 2011). Both sexes, but particularly males, are territorial and defend an area up to about 30 m from their nest (Muldal, Gibbs, & Robertson, 1985; Robertson & Rendell, 1990). Males arrive first on the breeding grounds to secure a nest site (i.e., a natural tree cavity or nest box), and most (94%–99%) are faithful to their previous breeding site (Winkler et al., 2011). Females, on the other hand, show a lower fidelity probability to their nest site (70%–94 %; Lagrange et al., 2014). Despite social monogamy, extra‐pair young are found in up to 90% of nests, compose on average about half of the young produced, and result from copulations occurring within 15 km from the nest (Dunn, Robertson, Michaud‐Freeman, & Boag, 1994; Kempenaers, Everding, Bishop, Boag, & Robertson, 2001; Lessard, Bourret, Bélisle, Pelletier, & Garant, 2014). The importance of extra‐pair fertilization in this species suggests that breeding site quality is related to breeder density not only as a source of information or competition, but also through the (extra‐pair) mating opportunities it provides. In our study area, females lay commonly 4–7 eggs and 65% of nests produce at least one fledgling with an average (±*SD*) of 4.0 ± 1.5 fledglings per successful nest (Ghilain & Bélisle, 2008). Once fledged, juveniles may explore nest sites for breeding in the following year (Chapman, 1935). There is little information about prospecting by adult tree swallows but it is assumed that subadult female floaters (second‐year nonbreeding females) gather information at the end of the breeding season (Stutchbury & Robertson, 1987). While prospecting, individuals can assess the density of both cavities and conspecifics as well as the level of breeding success experienced by conspecifics through the presence of nest material, eggs, young, feces, or dead nestlings in cavities (Nocera, Forbes, & Giraldeau, 2006). Suggestive evidence for the use of such information by tree swallows was found by Ghilain and Bélisle (2008) as well as by Robillard, Garant, and Bélisle (2013). Indeed, the likelihood that a nest box would be occupied in a given year increased with the fledging success that occurred in that box in the previous breeding season. Moreover, this result could not be explained by the philopatry of the previous occupants alone.

### 2.2 Study area

**Figure 1:**
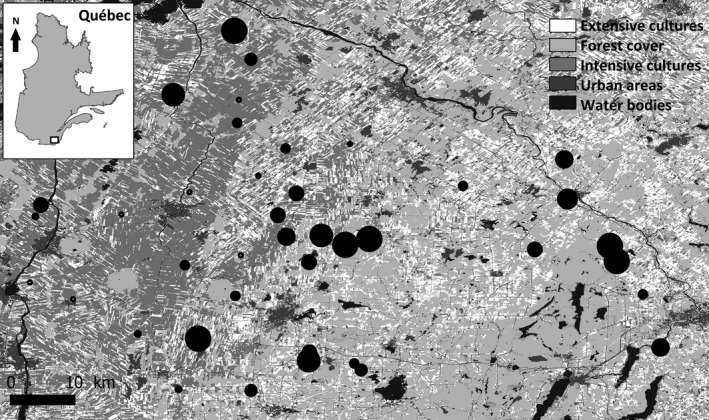
Distribution of the 40 farms where the breeding ecology of tree swallows was monitored between 2004 and 2013 in Southern Québec, Canada. The total number of fledglings produced on each farm during this period is depicted by the size of black dots (size fixed below 58 fledglings and proportional from 59 to 313 fledglings). Land cover types were based on a mosaic of classified Landsat‐TM satellite images (Canadian Wildlife Service, 2004)

We studied the breeding dispersal of female tree swallows within a network of 40 farms (hereafter sites) distributed within a 10,200‐km² gradient of agricultural intensification in southern Québec, Canada (see Ghilain & Bélisle, 2008 for additional details on the study system; Figure 1). Each site was separated from the nearest one by an average distance (±*SD*) of 7.28 ± 0.57 km. We have been monitoring closely the breeding activities of tree swallows on the 40 sites since 2004 and had banded 1595 breeding females by 2012. In this article, we included birds recaptured until 2013. Each site comprised 10 nest boxes aligned along a single field margin and spaced by 50 m to prevent territorial competition. The first and last nest boxes were thus separated by 450 m, the approximate distance up to which tree swallows are assumed to forage during the chick‐rearing period (McCarty & Winkler, 1999). Given this, we considered each cluster of 10 nest boxes as the spatial unit forming a colony for studying site fidelity. Between 2004 and 2013, an average of 38 ± 2 sites per year was occupied by at least one pair of tree swallows. Although we observe high rates of extra‐pair paternities in our study system, most of these paternities can be assigned to males breeding in our nest boxes within 15 km from focal nests, suggesting that there are few nondetected males breeding in nearby natural cavities or alternative nest boxes (Lessard et al., 2014).

### 2.3 Definition of RSi, RSc, and other variables

Previous empirical studies used either the number of fledglings produced by a female as a proxy of RSi (Beletsky & Orians, 1987; Pärt & Gustafsson, 1989; Schaub & Von Hirschheydt, 2009), or simply discretized that number into at least one fledgling (good RSi) or no fledgling (bad RSi) (Doligez, Danchin, Clobert, & Gustafsson, 1999; Naves, Monnat, & Cam, 2006). In our study area, females either failed to fledge any young (42.59% ± 14.01% (average ± *SD*) of females annually) or produced an average of 4.23 ± 1.41 fledglings per year between 2004 and 2013 (Appendix S1, Fig. S1). Given this bimodal frequency distribution of the annual number of fledglings produced per female, we coded RSi as zero versus at least one fledgling inasmuch as our capture–recapture approach requires that reproductive success be coded as a qualitative variable defining reproductive state. To eliminate potential biases due to intra‐annual dispersal on the estimate of interannual dispersal and the assessment of RSi, we excluded breeders of a second nesting attempt from our analyses, but not the additional public information left by these second nesting attempts (e.g., number of occupied nest boxes or produced fledglings). This decision was motivated by the fact that it would be very difficult to compare the yearly RSi of an individual that performed a single breeding attempt to one of an individual that made two attempts, the first of which had failed. Moreover, few studies, if any, have addressed how the history of intra‐annual breeding attempts and dispersal events impact interannual dispersal; leaving much uncertainty as to the weight that each breeding or dispersal attempt of a given year (given the number of attempts and their respective outcome) has in the decision process. Lastly, <10% of clutches originated from a second nesting attempt, implying (1) that intra‐annual breeding dispersal is likely of lesser importance to population structure and dynamics and (2) that we did not have enough data to address intra‐annual dispersal in a separate analysis.

We first assessed the RSc of a given site as the number of nest boxes that produced at least one fledgling within that site on a given year. A site was then categorized as being bad if its RSc was lower or equal to the median RSc calculated across all sites occupied by tree swallows on a given year. Alternatively, it was categorized as good if its RSc was greater than the population median. Note that results were not altered when we considered the reproductive success of an individual or not, when estimating the RSc experienced by that individual on a given year.

The RSc must be predictable from year to year to be used by individuals as an index of a site's reproductive quality based on information collected the previous year (Danchin et al., 1998). We therefore estimated the level of temporal autocorrelation in RSc across years based on the working correlation matrix of generalized estimating equations (GEEs; Agresti, 2002). Specifically, the annual RSc of sites (good/bad) was modeled as a constant in GEEs with a logit link function and binomial error structure. Given that RSc measures were spatially structured and longitudinal, the site acted as a clustering variable and the working correlation matrix was autoregressive. The GEEs were fitted in R 3.0.2 (R Development Core Team 2013) using the package geepack 1.1.6 (Halekoh, Højsgaard, & Yan, 2006).

To account for biases due to imperfect detection when estimating the effects of RSi and RSc on dispersal probabilities, we took into account the influence of the age and previous dispersal behavior of an individual on its likelihood to disperse using capture–recapture models. As the age of individuals is linked with reproductive success and has been found to affect dispersal in various bird species (Bouwhuis, Choquet, Sheldon, & Verhulst, 2012), including tree swallows (Steven, 1978), we distinguished females in their first breeding season (i.e., second‐year birds or SY) from potentially more experienced ones (i.e., after second‐year birds or ASY) based on plumage (Hussell, 1983). We also considered a “memory” effect (sensu Hestbeck, Nichols, & Malecki, 1991; equivalent to a prior dispersal effect) whereby individuals dispersing in the previous year may show a higher dispersal probability than individuals faithful to their breeding site at *t* – 1, as previously shown to occur in tree swallows from this system (Lagrange et al., 2014).

### 2.4 Capture–recapture analyses

#### 2.4.1 Definition of the multievent model

We used a multievent capture–recapture model (Pradel, 2005) adapted to study dispersal among numerous sites, as developed in Lagrange et al. (2014). Multievent models are used to estimate, between *t* and *t *+ 1, the probability to be faithful (to return on the same site two consecutive years) or to have dispersed (to change of site between two breeding seasons) even though an individual was not captured over two consecutive years (Pradel, 2005). We developed this model by integrating the RSc and RSi in the individual state. Our model comprised 25 states that conveyed information about the location of each individual (whether it occupied the same site as on the previous breeding/capture occasion or not) as well as information about whether the individual was captured or not on the previous and current breeding occasions (Appendix S2, Fig. S3). A capture status at *t *− 1 was required in the state because it partially conditions the event at *t* (e.g., see events 1 and 9, Appendix S2, Fig. S3) and, by construction of multievent capture–recapture models, only information present in the state at *t* may be used to predict the event at *t*. In turn, it is important to distinguish whether the capture status at *t* − 1 is known because then the dispersal status may be known. Information about RSi and RSc at *t* was added to the state to evaluate their impact on future dispersal. Note that when an individual was not captured at *t*, it did not matter whether it was captured at *t* − 1; in such cases, we did not have to specify a capture status at *t* − 1 for that individual. Thus, we retained 24 composite states and 13 events corresponding to the deducible field observations included in capture histories (Appendix S2, Fig. S3).

#### 2.4.2 Parameterization steps

Transitions between *t* and *t* + 1 involved five steps that gradually updated the information carried by the state. These steps allowed us to estimate parameters about apparent survival (S; as mortality cannot be distinguished from permanent emigration from the study area), fidelity (F), transition between RSc (C), transition between RSi (I), and recapture (R). Although the ultimate parameter of interest was F, the other “nuisance” parameters were considered in the model because they could indirectly affect the estimates of F. The matrix for each type of parameters is detailed in Appendix S2, Fig. S4. When first captured, the dispersal status of an individual cannot be known, but its RSi, RSc, and current capture status are. From the first state defining a captured bird, an individual can survive with probability (S), or die with probability (1 − S). When an individual survives at *t*, it can return at *t* + 1 to the same site it occupied at *t* with probability (F), or disperse to another site with probability (1 − F). The model then estimates the probability (C) that an individual experiences different RSc between *t* and *t* + 1, or the same RSc between these two breeding occasions (1 − C). Note that C is independent of F because an individual can be faithful to a given site between two consecutive years while the RSc of that site can change. Similarly, the model goes on to estimate the probability (I) that an individual obtains a different RSi between *t* and *t* + 1, or the same RSi between the two breeding occasions (1 − I). In the last step of the transition between *t* and *t* + 1, the probability of being captured (R) or not (1 − R) at *t* + 1 (corresponding to the suffix of the dispersal status; Appendix S2, Fig. S3) in each state is estimated. In the last matrix of our multievent model, we linked events and states. Note that one event could correspond to several states, but that each state could only correspond to a single event. Consequently, the probability of an event giving the state is either 0 or 1.

#### 2.4.3 Model selection and tested variables

We used goodness‐of‐fit (GOF) tests to assess the potential nonindependence of capture events for each individual in our dataset (e.g., transience and trap dependence effects). The overall lack of fit can be corrected in modeling, using the coefficient of overdispersion ĉ following Burnham and Anderson (2002). Standard errors, confidence intervals, and AIC values were adjusted for overdispersion, whenever detected. Because GOF tests are not yet developed for multievent models (Pradel, Gimenez, & Lebreton, 2005), we had to rely on those intended for standard capture–recapture unisite models and implemented in program U‐CARE (Choquet, Lebreton, Gimenez, Reboulet, & Pradel, 2009). This implied that we had to simplify events by using only recaptures (coded 1) or nonrecaptures (coded 0) of individuals (Sanz‐Aguilar et al., 2011).

Given the numerous potential model structures resulting from the large number of parameters, and of states and variables potentially influencing those parameters, we used a model selection procedure that established a model structure one step at a time in order to reduce model selection uncertainty. Because no study has examined the movement of tree swallows in a capture–recapture context before, we opted to establish model structures starting with the parameterization step having the least relevance to the one having the most relevance regarding the questions addressed by our study, namely R, C, I, S, and F. We defined a list of competing models for each step where parameters may vary with the effects of prior dispersal (memory effect), RSi, RSc, or a combination of those as main effects or interactions, as well as the effects of time or age as main effects (Appendix S3). For instance, starting with parameters in step R, we compared competing models whose structure varied in line with specific predictions (see Table 1), while keeping the other parameters (i.e., those included in steps C, I, S, and F) constant. The “best” model from this initial list was identified as the one showing the lowest Akaike information criterion corrected for small samples and overdispersion (QAIC_c_; Burnham & Anderson, 2002). The initial list of models was then augmented by adding time or age effects to the best model from that list (Appendix S3, Table S1). The “best” model structure from the extended list of competing models was finally retained for R before repeating the same procedure with the next step of interest, here C. When the best model structure had been found for all parameterization steps, we conducted a second round of model selection whereby the evidence relative to the hypotheses concerning parameters included in step S, and then I, was reassessed (Table 1), but this time with the model structures identified in the previous round of model selection (Appendix S3, Table S2). This second round allowed calculating the Akaike weight for each model *i* (*w*_*i*_) having a structure already determined for all parameters (i.e., all models of the second round and models of step F of the first round; Table 2). In this way, *w*_*i*_ were not affected by the constant structure of a nondefined step in the first round of model selection. We present model‐averaged parameter estimates and their unconditional standard errors (noted ± *SE*) based on the QAIC_c_ and *w*_*i*_ (≥0.01) of candidate models (Burnham & Anderson, 2002). All models were implemented in program E‐SURGE (Choquet, Rouan, & Pradel, 2009).

**Table 1:** Predictions about breeding dispersal of female tree swallows tested using our multievent capture–recapture model with respect to site fidelity (F) and nuisance parameters regarding recapture (R), transition between a good/bad conspecific reproductive success (C), transition between a good/bad individual reproductive success (I), and survival (S)

Parameters	Effects	Predictions
R	*i*	All states have the same recapture probability.
	‘RSi’	Recapture is higher if a female is successful at producing fledglings because there are more occasions to capture her.
	*t*	Recapture success varies over time (years).
C	*i*	Transition probability of experimenting a bad and then a good RSc is equal to that of experimenting a good and then a bad RSc.
	‘RSc’	Transition probability of experimenting a bad and then a good RSc differs from that of experimenting a good and then a bad RSc.
	*t*	Transition probability varies over time but with the same probability in both directions.
I	*i*	Transition probability of experimenting a bad and then a good RSi is equal to that of experimenting a good and then a bad RSi.
	‘RSi’	RSi varies from one year to another.
	‘RSc’*’RSi’	Females with a bad RSi at *t* are more likely to experience a good RSi at *t *+ 1 when RSc at *t* is good than when it is bad.
	‘memory’*’RSi’	1) Assuming that dispersal results from a bad RSi, dispersers should improve their RSi compared to females faithful to their breeding site.
		2) Assuming that dispersal is costly, dispersers with a good RSi at *t* are more likely to experience a bad RSi at *t *+ 1 than females faithful to their breeding site.
	t	Transition probability varies over time (years) but with the same probability in both directions.
	age	Young (SY) females without experience are more likely to transit from a bad to a good RSi than older (ASY) females.
S	*i*	All states have the same apparent survival probability.
	‘memory’	Dispersing females have a lower probability to survive than faithful ones because they are likely exposed to greater risks.
	‘RSi’	1) Assuming that RSi is a proxy of individual condition, females with a good RSi should be more likely to survive than females with a bad RSi.
		2) Assuming that RSi is a proxy of breeding costs, female with a good RSi should be less likely to survive than females with a bad RSi.
	*t*	Apparent survival varies over time (years).
	age	SY females should be less likely to survive than ASY females because they lack experience with respect to the challenges and costs they may face during breeding, migration or winter.
F	*i*	All states have the same dispersal probability.
	‘memory’	Females dispersing at *t *− 1 should be more likely to disperse at *t* than females that were faithful to their breeding site at *t *− 1.
	‘RSi’	Females experiencing a good RSi should be more faithful to their breeding site than females experiencing a bad RSi.
	‘RSc’	Females breeding on a site with a bad RSc should be more likely to disperse than females breeding on a site with a good RSc.
	*t*	Dispersal probability varies over time (years).
	age	SY females should be more likely to disperse than ASY females.

**Table 2:** Model selection examining the effect of individual (RSi) and conspecific reproductive success (RSc), prior dispersal (memory), age (SY vs. ASY), and time (year) on the parameters of the multievent capture–recapture model fitted for female tree swallows breeding in Southern Québec, Canada, 2004–2013. Model parameters estimate the probability of recapture (R), transition between a good/bad conspecific reproductive success (C), transition between a good/bad individual reproductive success (I), survival (S), and site fidelity (F). Only the models used for model averaging (*w*_*i*_ ≥ 0.01) are shown

Models	*k*	ΔQAIC_c_	*w*_*i*_	#
S(RSi) F(RSi*memory) I(RSi*RSc) C(RSc) R(RSi)	22	0.00	0.36	55
S(RSi*memory) F(RSi*memory) I(RSi*RSc) C(RSc) R(RSi)	24	0.91	0.23	S44
S(RSi*age) F(RSi*memory) I(RSi*RSc) C(RSc) R(RSi)	24	1.95	0.14	S49
S(RSi) F(RSi*memory*age) I(RSi*RSc) C(RSc) R(RSi)	24	2.48	0.10	63
S(RSi) F(RSi*memory+*t*) I(RSi*RSc) C(RSc) R(RSi)	30	3.02	0.08	61
S(RSi) F(RSi*memory) I(RSi*RSc*memory) C(RSc) R(RSi)	26	3.83	0.05	I30
S(RSi+*t*) F(RSi*memory) I(RSi*RSc) C(RSc) R(RSi)	30	5.84	0.02	S47
S(RSi) F(RSi*RSc*memory) I(RSi*RSc) C(RSc) R(RSi)	26	7.22	0.01	58
S(RSi) F(RSi) I(RSi*RSc) C(RSc) R(RSi)	18	7.47	0.01	53

Other parameters conserved the basic structure included an effect of RSi on R, and no effect on C (as in #55, Appendix S3, Table S1). For S, F, and I, the effects were tested alone, in addition (+) or in interaction (*). k is the number of model parameters used to calculate ΔQAIC_c_ (ĉ = 1.94; Model_55_ showed the lowest QAIC_c _= 4265.80) and corresponding Akaike weight (*w*_*i*_). # is the number of the model (see Appendix S3, Tables S1 and S2 for a full list of models).

## 3 RESULTS

Between 2004 and 2013, an average (±*SD*) of 4.61 ± 0.78 nest boxes per site produced at least one fledgling on a yearly basis and between 38% and 53% of sites showed a bad RSc depending on year. RSc was strongly and positively autocorrelated across years (*r* = .70 ± 0.06). This result supports the assumption that RSc was predictable in space and time and thus could potentially act as a determinant of dispersal decisions in our system.

### 3.1 Goodness‐of‐fit of capture–recapture model

Goodness‐of‐fit tests indicated a lack of fit of the general model that considered all transitions possible between states over time when fitted to the data (χ² = 68.22, *df* = 35, *p* < .001). Although no trap dependence was detected, a transience effect was found, especially for SY females (χ² = 30.04, *df* = 6, *p* < .001). Because apparent survival was lower for SY than for ASY females (χ² = 21.19, *df* = 7, *p* = .03), SY females were less likely to be recaptured than ASY ones. We corrected for the overall lack of fit by using a variance inflation factor ĉ = 1.94.

### 3.2 Model selection

The basic parameter structure obtained through the first selection indicated an effect of RSi on R, S, and F, an effect of RSc on I, a prior dispersal (memory) effect on F, and no time or age effects (Appendix S3, Table S1). Results of the “nuisance” parameters (R and C) from the first selection are as follows.

#### 3.2.1 First round: Estimates of R and C

##### Recapture (R)

The model including an RSi effect (model_14_) showed a QAIC_c_ 52.7 points lower than the constant model (model_11_). As expected, a female with a good RSi was recaptured with a higher probability (0.99 ± 0.00) than a female with a bad RSi (0.32 ± 0.06). These probabilities appeared constant across years (∆QAIC_c_ for model_18_ vs. model_14_ = 4.68).

##### Transition between RSc (C)

The probability that an individual experienced a good RSc after having experienced a good RSc in the previous breeding seasons was (0.81 ± 0.03), which is nearly twice as high as the probability that it experienced a bad RSc given that it had experienced a bad RSc in the previous breeding seasons (0.44 ± 0.06). Time had no effect on this parameter (∆QAIC_c_ for model_22_ vs. model_20_ = 12.26).

#### 3.2.2 Second round: Estimates of I, S, and F

The second round of model selection (Appendix S3, Table S2) was initiated based on the “best” model identified in the first round of selection, namely model_55_ (QAIC_c_ = 4,265.80). None of the models considered in that second round performed better than model_55_ (*w*_55_ = 0.36), thereby lending support to the parameter structure obtained for F in the first round. Few models obtained an empirical support equivalent (i.e., *w*_*i*_ > 0.05) to that of model_55_ (Table 2).

##### Transition between RSi (I)

**Figure 2:**
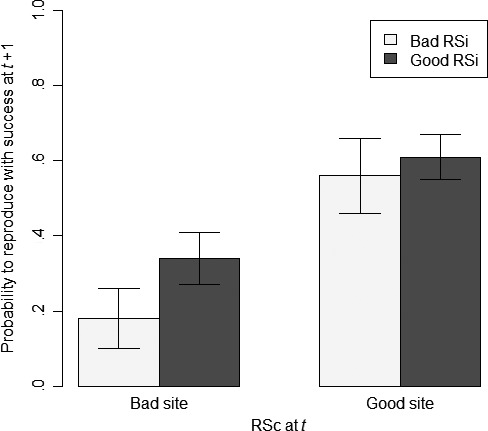
Probability that female tree swallows reproduced with success (i.e., fledged at least one young at *t* + 1) as a function of their previous individual reproductive success (RSi) and according to whether they bred on a site with a good or a bad conspecific reproductive success (RSc) on their previous breeding occasion (i.e., at *t*). Estimates and their unconditional *SE* stem from model‐averaged parameters

The likelihood that females kept the same RSi in two consecutive years varied with the RSc experienced in the first of these two breeding events, as indicated by all of the models that were retained after the second round of model selection (Table 2). Dispersal behavior in the previous year was, however, not a clear determinant of I (*w*_I30_ **= 0.05; Table 2). After breeding on a site characterized by a good RSc, females were less likely to keep a bad RSi in the second of two consecutive years (0.44 ± 0.10) than those that bred on a site with a bad RSc (0.82 ± 0.08; Figure 2). Conversely, females had a higher probability of keeping a good RSi after breeding on a site that showed a good RSc (0.62 ± 0.05) than after breeding on a site with a bad RSc (0.34 ± 0.07).

##### Survival (S)

**Figure 3:**
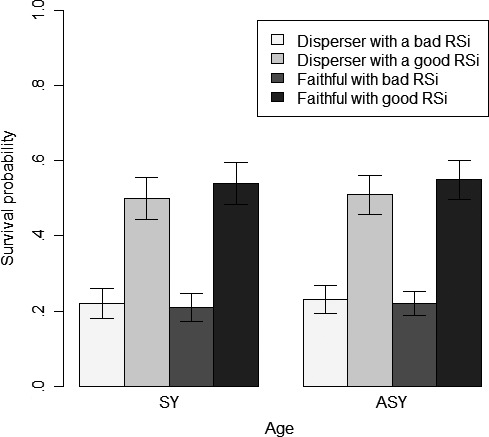
Apparent survival of female tree swallows between two consecutive years according to their age (SY: second year vs. ASY: after second year), prior dispersal behavior, and individual reproductive success. Estimates and their unconditional *SE* stem from model‐averaged parameters

Females obtaining a good RSi were more than twice as likely to survive to the next breeding season (or not permanently emigrate) than females with a bad RSi (Figure 3). Although the influence of RSi on apparent survival probability may depend on the age of females (*w*_S49_ = 0.14; Table 2), this dependency was marginal and SY females showed a survival probability only 0.01 lower than ASY females (Figure 3). However, the dispersal behavior exhibited by females in the previous year modulated the influence of RSi on apparent survival probabilities (*w*_S44_ = 0.23; Table 2). Indeed, females that obtained a good RSi at *t* after dispersing in the previous year were slightly less likely to survive to the next breeding season (or not permanently emigrate) than faithful ones (SY: 0.50 ± 0.06 vs. 0.54 ± 0.06; ASY: 0.51 ± 0.05 vs. 0.54 ± 0.05). On the other hand, when they had a bad RSi, they survived equally well (albeit at a much lower probability) whether they had dispersed in the previous year or not (SY: 0.22 ± 0.04 vs. 0.21 ± 0.04; ASY: 0.23 ± 0.04 vs. 0.22 ± 0.03). Although a time effect was included in a model selected for model averaging (i.e., *w*_S47_ = 0.02; Table 2), apparent survival probability estimates barely varied across years.

##### Fidelity (F) and Dispersal

**Figure 4:**
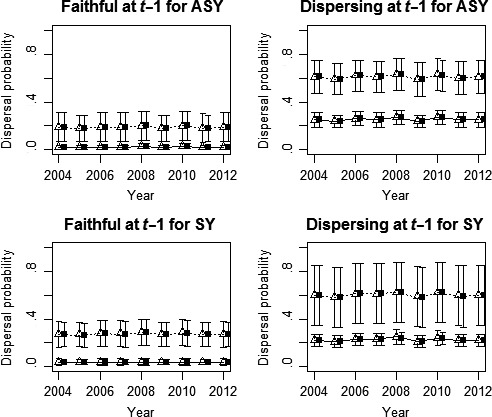
Dispersal probabilities of female tree swallows as a function of their age (SY: second year vs. ASY: after second year), prior dispersal behavior, and individual reproductive success (RSi), as well as according to whether they bred on a site with a good or bad conspecific reproductive success (RSc). RSi appears in lines (continuous for good RSi, dotted for bad RSi), RSc in the form of point (empty triangles for good RSc, black squares for bad RSc). Estimates and their unconditional *SE* stem from model‐averaged parameters

All but one of the models (i.e., model_53_) in the multimodel inference provided support for the hypothesis that dispersal probabilities are affected by RSi and that this effect depends upon the dispersal behavior of the female in the previous year (Table 2). In contrast, only one model suggested that RSc could affect dispersal probabilities (*w*_58_ = 0.01; Table 2). Age and time effects were only contained in models that received moderate support from the data (age: *w*_63_ = 0.10 and time: *w*_61_ = 0.08; Table 2). According to model‐averaged parameters, females that experienced a bad RSi after having dispersed in the previous year were 30 times more likely to disperse than faithful females that obtained a good RSi (0.61 ± 0.13 vs. 0.02 ± 0.02; Figure 4). Females with a bad RSi that did not disperse in the previous year and females with a good RSi that dispersed in the previous year showed similar and intermediate dispersal probabilities (0.19 ± 0.12 vs. 0.24 ± 0.05). Dispersal probabilities were relatively constant across years, and age did not affect the probability of dispersal (Figure 4).

## 4 DISCUSSION

Our results agree with the plethora of empirical studies suggesting that female birds are more likely to disperse after experiencing a breeding failure, a behavior expected to improve the odds of finding a better mate or breeding site for the next breeding event (Greenwood & Harvey, 1982; Hoover, 2003; Johnson & Gaines, 1990). On the other hand, we found no evidence to suggest that female birds modulate their decision to disperse on the basis of the breeding success of surrounding conspecifics (RSc). Yet both theoretical and empirical work suggested that reproductive failure of conspecifics should promote dispersal, especially when individuals experience a breeding failure themselves and (site‐specific) RSc is temporally autocorrelated (Boulinier & Danchin, 1997; Danchin et al., 1998). Given that our study system met this latter condition and that we used a capture–recapture approach that limits potential biases when estimating dispersal probabilities (unlike previous studies that assessed RSc effects on dispersal; e.g., Doligez et al., 1999; Serrano et al., 2001), our results suggest that the value and use of RSc as public information to guide dispersal decisions are likely dictated by multiple ecological determinants if this cue is indeed used. For instance, public information should be most useful when individual sampling is costly and/or when the time available to find and exploit resources is strongly limited (Clark & Mangel, 1986). The use of public information for dispersal may thus depend on landscape structure which partly dictates the spatial distribution and density of both breeding sites and conspecifics (i.e., potential mates, competitors, and sources of information; Doligez et al., 2003) as well as movement (prospection) costs (Bélisle, 2005). Moreover, the value of public information when selecting a breeding site may depend upon individual factors such as age or experience (Nocera, Forbes, & Giraldeau, 2009; Nocera et al., 2006). Understanding the influence of such factors on the value of public information is crucial as this type of information can be available but not used by individuals (Racine, Giraldeau, Patenaude‐Monette, & Giroux, 2012).

Female tree swallows that failed to fledge at least one young were 2.5 (=0.61/0.24) to 9.5 (=0.19/0.02) times more likely to disperse than females that succeeded at fledging at least one young, depending on whether they dispersed or not in the previous year, respectively (Figure 4). Our results contrast with those of Shutler and Clark (2003) showing that tree swallows breeding dispersal among 115 nest boxes separated between 30 m and 3.8 km from one another (<1 km between nest box clusters) was not driven by RSi (defined as the number of fledglings). Such a discrepancy may result from the fact that these authors considered dispersal as a change of nest boxes between two consecutive breeding events at a much smaller spatial scale than within our multisite system. Moreover, Shutler and Clark (2003) manipulated RSi either by increasing or by decreasing the clutch size of females and may thereby have affected the perception of reproductive success by females. On the other hand, our results agree with those of Winkler et al. (2004) who worked in a multisite context within an heterogeneous landscape, but at a much smaller spatial extent than our study system (6 main sites of 22‐131 nest boxes each and <25 km apart; neighboring boxes spaced by 20 m). They observed that females failing to fledge any young dispersed more often than those that bred successfully (i.e., 28% vs. 5%). Such methodological and conclusion discrepancies among studies about dispersal are not restricted to tree swallows as they are also found in other species (e.g., Montalvo & Potti, 1992; Paradis, Baillie, Sutherland, & Gregory, 1998). This certainly stresses the importance that future studies take landscape structure (including the spatial distribution of available territories or nest sites), grain, and extent into account when defining what constitutes a dispersal event and thus, when determining at which spatial scale it occurs and how it is affected by RSi.

The lack of effect of RSc on dispersal we observed contrasts with the influence that this variable had on the breeding dispersal probability of another passerine, the collared flycatcher, *Ficedula albicollis*, which is a second‐order cavity nester just like the tree swallow. Indeed, low RSc promoted dispersal defined as a change of woodland between successive breeding events in females of this forest bird (Doligez et al., 1999). Although both species are migratory and defend territories restricted to the immediate surroundings of their nest sites, tree swallows differ from collared flycatchers by being semicolonial (Robertson, Gibbs, & Stutchbury, 1986; Winkler et al., 2011). This latter aspect, however, is unlikely to explain the difference between our results and those of Doligez et al. (1999) as the other studies that found an effect of RSc on dispersal involved the colonial black‐legged kittiwakes (Danchin et al., 1998) and lesser kestrels, *Falco naumanni* (Serrano et al., 2001). An alternative explanation may reside in the fact that swallows are much more mobile than flycatchers during the breeding season. SRc and its effect on dispersal may indeed be easier to assess in species with small neighborhoods (sensu Addicott et al., 1987) than in species that can foray and collect information over large areas. The lack of RSc effect on dispersal we observed is also surprising given that the different breeding sites varied (substantially) in relative quality and that site quality was predictable to some degree across breeding events, two conditions that must be met for RSc to influence dispersal (Danchin et al., 1998; Doligez et al., 1999). Yet, the quality of the best breeding sites was more predictable than that of poor ones (Appendix S1, Fig. S2), and such variation in predictability of site quality is expected to reduce the value of RSc as reliable public information (Switzer, 1993, 1997). This being said, how much and under what circumstances unpredictability in site quality is sufficient to impair decision making with respect to dispersal are currently unknown (Doligez et al., 2003; Lecomte, Gauthier, & Giroux, 2008). The same could be said about the influence of environmental predictability on the value and reliability of both personal and public information and how these two types of information are integrated and used, especially outside foraging contexts (Pärt, Arlt, Doligez, Low, & Qvarnström, 2011; Rieucau & Giraldeau, 2011). These aspects should definitely be addressed by future research for a better appraisal of the (context‐dependent) importance of RSi and RSc in determining dispersal decisions.

We defined RSc based on the number of nest boxes that produced at least one fledgling within a given site in a given year, a measure that turned out to be associated with the density of breeders on the site (*r* = .80). This definition can be an indicator of expected number of potential sexual partners and reproductive success, inasmuch as dominant or healthy individuals should tend to gather in good sites, but also provides information about expected levels of competition or other detrimental effects such as parasitism (Brown and Brown 2000; Doligez et al., 1999). Density‐dependent effects may have biased our results on female breeding dispersal is unclear. Indeed, although high breeder density has been found to promote dispersal in males and site fidelity in females of some species (e.g., Doligez et al., 1999), no clear general trend of density‐dependent dispersal has been found for birds (Matthysen, 2005). Annual nest box occupancy in our study system (61% ± 15%) was such that intraspecific nest‐site competition is unlikely to have played a significant role, as it was exceptional that all nest boxes of a given site were occupied on any given year (Robillard et al., 2013). Also, defining the RSc based on the proportion of nest boxes that produced at least one fledging by site (good site = proportion higher or equal to the yearly median) instead of on the number of nest boxes that fledged at least one young did not affect the decision of tree swallows to disperse or not (see model selection in Appendix S3, Table S3). This being said, it appears clear that more studies focusing on the potential cues used by individuals to base their dispersal decisions are needed, especially given that these cues and effects may vary according to the species, sex, and condition of individuals as well as in a nonlinear fashion with breeder density (Doligez et al., 2003; Nocera et al., 2006).

The influence that RSc can have on dispersal decision making is likely modulated by movement constraints that could restrict site prospecting. Indeed, travel costs, and thereby distance among resource patches, have the potential to impede movements and in turn disrupt habitat sampling and selection (Beauchamp, Belisle, & Giraldeau, 1997; Bélisle, 2005; Bernstein, Kacelnik, & Krebs, 1988). In our system, sites were distant from one another by 42.2 ± 21.1 km on average (pairwise mean distance ± *SD*), and the distance to the nearest site averaged 7.3 ± 3.5 km, with potentially few alternative, natural or artificial breeding sites surrounding those provided by our nest box network (as suggested by the fact that males from our nestbox network sire >75% of the young (Lessard et al., 2014) and from point counts performed in our study area (M. Bélisle, unpublished data)). Under such conditions, tree swallows may not have had the opportunity to compare the quality of their breeding site with that of several other sites, especially given that they initiate their southward migration rapidly after fledging their young (Burke, 2014). Although tree swallows are vagile early in the breeding period (Dunn & Whittingham, 2005; Lessard et al., 2014), distance between colonies and the short time before the onset of the fall migration could explain why the site‐specific proportion of nest boxes producing at least one fledgling did not affect their dispersal decision. Studies showing the adaptive advantage of prospecting and public information use as means to improve breeding success through dispersal are accumulating (Badyaev, Martin, & Etges, 1996; Dittmann et al., 2005; Pärt & Doligez, 2003; Pärt et al., 2011; Schjørring, Gregersen, & Bregnballe, 1999). However, how landscape structure (i.e., the spatial distribution of potential breeding sites and the composition and configuration of intervening habitats) and the travel costs it may impose on individuals affect dispersal decisions is a research area still in its infancy (Stamps, Krishnan, & Reid, 2005; Zollner & Lima, 2005). Documenting prospective movements of individuals, especially those showing breeding difficulties or failure, with the help of modern tracking devices shall nevertheless help us push the envelope further (Ponchon et al., 2013).

Although RSc did not affect the decision to disperse, it may have influenced the settlement decision of female tree swallows. Indeed, 69% of the females were initially captured on a good site (based on RSc). Moreover, the probability that a female bred on a good site then on a bad one in subsequent years was lower than the probability it consecutively bred on a good site (0.19 vs. 0.81 ± 0.03; the yearly proportion of bad sites varied between 38% ± 16.1 and 53% ± 16.6). Analogously, the probability that a female bred on a bad site then on a good one was higher than the probability it consecutively bred on a bad site (0.56 vs. 0.44 ± 0.06). Also, females that settled on a site where conspecifics experienced a good success were twice as likely to have a good RSi as females that settled on a bad site. Such settlement patterns toward sites that produce the greater numbers of fledglings have been observed in other passerines (Brown et al., 2000; Doligez et al., 2002) and provide evidence that they result from an adaptive response of individuals to improve their fitness (Bowler & Benton, 2005).

Using a simpler model, Lagrange et al. (2014) showed the presence of individual heterogeneity in dispersal propensity within the same population of tree swallows. Yet, our results not only showed that some individuals appeared to have a greater dispersal propensity than others, and they also showed that this tendency was modulated by their breeding experience. Indeed, the likelihood that a female dispersed after having dispersed in the previous year was 0.19 and 0.61 depending if she experienced a good or a bad RSi, respectively, and 0.02 and 0.24 if she was site faithful in the previous year. Moreover, our results suggested that this individual variation in dispersal propensity likely increases with age as females that showed site fidelity after breeding in their second year became more faithful in following years (+1.8% to +8.5% between SY and ASY), whereas females that dispersed after breeding in their second year were more inclined to disperse later on in life (+0.8% to +2.7% between SY and ASY). These patterns support the hypothesis that dispersal propensity may not only depend on individual experience, but also on phenotype, a condition that can have important implications for the dynamics of spatially structured populations (Clobert, Galliard, Cote, Meylan, & Massot, 2009; Cote, Clobert, Brodin, Fogarty, & Sih, 2010; Leimar & Norberg, 1997). Given the ever‐increasing habitat loss and fragmentation of natural habitats, the study of individual heterogeneity in dispersal‐related traits should certainly receive more attention (Cote et al., 2017).

Dispersal may involve a variety of costs including time, energy, predation risk, and opportunity losses that can be incurred prior, during, or after it occurred (Yoder, Marschall, & Swanson, 2004; reviewed in Bonte et al., 2012). Our results suggest that tree swallows are not exempt from such costs regarding dispersal or other ecological processes such as migration. For instance, SY and ASY females that experienced a good RSi after having dispersed in the previous year had a survival reduction (or permanent emigration increase) of 4% compared to females that did not change location. The fact that this pattern was, however, not observed in females that experienced a bad RSi (and thus did not fledge any young and thereby probably invested less in reproduction) points toward a combined effect of dispersal and raising a brood on survival. Still, our results did not indicate that dispersal influenced the RSi of females. Yet, our RSi index may have been defined too crudely for detecting an effect of dispersal on subsequent reproductive output. Pursuing the development of capture–recapture models that allow the incorporation of nominal ordinal or continuous “covariates”, such as site isolation or reproductive success, is certainly warranted, especially given the importance of dispersal costs on the evolution and form of this behavior (Johnson & Gaines, 1990).

Our results showed a detection three times higher for females breeding successfully than for those experiencing a bad RSi. Females with breeding failure spent less time on their clutch or brood and the occasions to capture them were hence limited. Such heterogeneity has the potential to influence estimates of dispersal probabilities. Previous studies that found an effect of RSc on dispersal probabilities assumed a perfect detection of individuals. Applying a similar approach to our data (Appendix S4), models that included RSc and its interaction with RSi were found to be the most parsimonious (Table S4) even though the effect of RSc was nonsignificant (Table S5). Globally, dispersal probabilities estimated with GLMMs depicted the same trends as those found with capture–recapture models (Appendix S4, Fig. S5). Yet, how dispersal was defined affected the estimates obtained with GLMMs. When the dataset was composed of individuals captured in two consecutive years, dispersal probabilities were underestimated compared to those obtained by capture–recapture models, but were overestimated when the dataset included individuals captured at least twice but not necessarily in two consecutive years. Assuming a perfect detection thus appear more likely to bias dispersal estimates and the effect of covariates on those estimates than multievent capture–recapture models, which can include “nuisance” parameters to avoid confounding effects. The capture–recapture approach used in this paper partitioned the effects of variables through a series of matrices for each nuisance parameter: Recapture (R) and fidelity were conditional on RSi, and transitions between good/bad RSi (I) were linked to RSc. With a GLMM approach, nuisance parameters (e.g., R or I) are absent and variables affecting them have the potential to act directly on F. This shortcut has the potential to bias conclusions and therefore advocates the systematic consideration of detection probabilities in future dispersal studies. It is only by limiting biases and replicating studies across species showing different life histories and subject to diverse ecological conditions that we will obtain a clearer understanding of the respective roles of RSi and RSc in dispersal decisions. Our capture–recapture approach based on Lagrange et al. (2014) offers great opportunities to undertake such endeavor as it can be easily extended and adapted to a variety of ecological questions and taxa. For example, Cayuela, Pradel, Joly, and Besnard (2017) adapted Lagrange's et al. model to assess the influence of habitat (pond) type (a site‐specific characteristic) on the apparent survival and (between‐pond) movement probability of toads of different ages and sex.

## CONFLICT OF INTEREST

None declared.

## AUTHOR CONTRIBUTIONS

Paméla Lagrange collected a part of long‐term data, realized analyses and modeling, and wrote the article. Marc Bélisle and Olivier Gimenez supervised the analyses and the redaction. Roger Pradel supervised the modeling step (analyses and redaction). Dany Garant, Fanie Pelletier, and Marc Bélisle provided the financial support for field work and data collection. Blandine Doligez, Dany Garant, and Fanie Pelletier contributed to write the article.

## References

[ece33241-bib-0001] Addicott, J. F. , Aho, J. M. , Antolin, M. F. , Padilla, D. K. , Richardson, J. S. , & Soluk, D. A. (1987). Ecological neighborhoods: Scaling environmental patterns. Oikos, 49, 340–346.

[ece33241-bib-0002] Agresti, A. (2002). Categorical data analysis. Hoboken, NY: John Wiley & Sons Inc.

[ece33241-bib-0003] Badyaev, A. V. , Martin, T. E. , & Etges, W. J. (1996). Habitat sampling and habitat selection by female wild turkeys: Ecological correlates and reproductive consequences. Auk, 13, 636–646.

[ece33241-bib-0004] Beauchamp, G. , Belisle, M. , & Giraldeau, L. A. (1997). Influence of conspecific attraction on the spatial distribution of learning foragers in a patchy habitat. Journal of Animal Ecology, 66, 671–682.

[ece33241-bib-0005] Beletsky, L. D. , & Orians, G. H. (1987). Territoriality among male red‐winged blackbirds. Behavioral Ecology and Sociobiology, 20, 21–34.

[ece33241-bib-0006] Bélisle, M. (2005). Measuring landscape connectivity: The challenge of behavioral landscape ecology. Ecology, 86, 1988–1995.

[ece33241-bib-0007] Bernstein, C. , Kacelnik, A. , & Krebs, J. R. (1988). Individual decisions and the distribution of predators in a patchy environment. II. The influence of travel costs and structure of the environment. Journal of Animal Ecology, 57, 1007–1026.

[ece33241-bib-0008] Betts, M. G. , Hadley, A. S. , Rodenhouse, N. , & Nocera, J. J. (2008). Social information trumps vegetation structure in breeding‐site selection by a migrant songbird. Proceedings of the Royal Society of London. Series B, Biological Sciences, 275, 2257–2263. 18559326 10.1098/rspb.2008.0217 PMC2603235

[ece33241-bib-0009] Bonte, D. , Van Dyck, H. , Bullock, J. M. , Coulon, A. , Delgado, M. , Gibbs, M. , … Travis, J. M. J. (2012). Costs of dispersal. Biological Reviews, 87, 290–312. 21929715 10.1111/j.1469-185X.2011.00201.x

[ece33241-bib-0010] Boulinier, T. , & Danchin, E. (1997). The use of conspecific reproductive success for breeding patch selection in terrestrial migratory species. Evolutionary Ecology, 11, 505–517.

[ece33241-bib-0011] Boulinier, T. , McCoy, K. D. , Yoccoz, N. G. , Gasparini, J. , & Tveraa, T. (2008). Public information affects breeding dispersal in a colonial bird: Kittiwakes cue on neighbours. Biology Letters, 4, 538–540. 18647711 10.1098/rsbl.2008.0291 PMC2610090

[ece33241-bib-0012] Bouwhuis, S. , Choquet, R. , Sheldon, B. C. , & Verhulst, S. (2012). The forms and fitness cost of senescence: Age‐specific recapture, survival, reproduction, and reproductive value in a wild bird population. American Naturalist, 179, E15–E27. 10.1086/663194 22173469

[ece33241-bib-0501] Bowler, D. E. , & Benton, T. G. (2005). Causes and consequences of animal dispersal strategies: Relating individual behaviour to spatial dynamics. Biological Reviews, 80, 205–225. 15921049 10.1017/s1464793104006645

[ece33241-bib-0013] Brown, C. R. , & Brown, M. B. (1986). Ectoparasitism as a Cost of Coloniality in Cliff Swallows (Hirundo Pyrrhonota). Ecology, 67, 1206–1218.

[ece33241-bib-0500] Brown, C. R. , Brown, M. B. , & Danchin, E. (2000). Breeding habitat selection in cliff swallows: The effect of conspecific reproductive success on colony choice. Journal of Animal Ecology, 69, 133–142.

[ece33241-bib-0015] Burke, L. (2014). Migration and carry‐over effects in tree swallows (Tachycineta bicolor). Master thesis, Dalhousie University, Nova Scotia.

[ece33241-bib-0016] Burnham, K. P. , & Anderson, D. R. (2002). Model selection and multimodel inference: A practical information‐theoretic approach. New York, NY: Springer.

[ece33241-bib-0017] Calabuig, G. , Ortego, J. , Aparicio, J. M. , & Cordero, P. J. (2008). Public information in selection of nesting colony by lesser kestrels: Which cues are used and when are they obtained? Animal Behavior, 75, 1611–1617.

[ece33241-bib-0018] Cam, E. , & Monnat, J. Y. (2000). Apparent inferiority of first‐time breeders in the kittiwake: The role of heterogeneity among age classes. Journal of Animal Ecology, 69, 380–394.

[ece33241-bib-0019] Canadian Wildlife Service (2004). Occupation du sol à partir des images classifiées Landsat‐7, Sud du Québec, 1999–2003. Environnement Canada, région du Québec, Québec, Canada.

[ece33241-bib-0020] Cayuela, H. , Pradel, R. , Joly, P. and Besnard, A. (2017). Analysing movement behaviour and dynamic space‐use strategies among habitats using multi‐event capture‐recapture modelling. Methods in Ecology and Evolution, doi:10.1111/2041‐210X.12717

[ece33241-bib-0021] Chapman, L. B. (1935). Studies of a tree swallow colony. Bird‐Banding, 26, 45–57.

[ece33241-bib-0022] Choquet, R. , Lebreton, J. D. , Gimenez, O. , Reboulet, A. M. , & Pradel, R. (2009). U‐CARE: Utilities for performing goodness of fit tests and manipulating CApture–REcapture data. Ecography, 32, 1071–1074.

[ece33241-bib-0023] Choquet, R. , Rouan, L. , & Pradel, R. (2009). Program E‐SURGE: A software application for fitting multievent models. Environmental and Ecological Statistics, 3, 845–865.

[ece33241-bib-0024] Clark, C. W. , & Mangel, M. (1986). The evolutionary advantages of group foraging. Theoretical Population Biology, 30, 45–75.

[ece33241-bib-0025] Clobert, J. , Danchin, E. , Nichols, J. D. , & Dhondt, A. A. (2001). Dispersal. New York, NY: Oxford University Press.

[ece33241-bib-0026] Clobert, J. , Galliard, L. , Cote, J. , Meylan, S. , & Massot, M. (2009). Informed dispersal, heterogeneity in animal dispersal syndromes and the dynamics of spatially structured populations. Ecology Letters, 12, 197–209. 19170731 10.1111/j.1461-0248.2008.01267.x

[ece33241-bib-0027] Cote, J. , Bestion, E. , Jacob, S. , Travis, J. , Legrand, D. , & Baguette, M. (2017). Evolution of dispersal strategies and dispersal syndromes in fragmented landscapes. Ecography, 40, 56–73.

[ece33241-bib-0028] Cote, J. , Clobert, J. , Brodin, T. , Fogarty, S. , & Sih, A. (2010). Personality‐dependent dispersal: Characterization, ontogeny and consequences for spatially structured populations. Philosophical Transactions of the Royal Society of London. Series B, Biological Sciences, 365, 4065–4076. 21078658 10.1098/rstb.2010.0176 PMC2992741

[ece33241-bib-0029] Dall, S. R. , Giraldeau, L.‐A. , Olsson, O. , McNamara, J. M. , & Stephens, D. W. (2005). Information and its use by animals in evolutionary ecology. Trends in Ecology & Evolution, 20, 187–193. 16701367 10.1016/j.tree.2005.01.010

[ece33241-bib-0030] Danchin, E. , Boulinier, T. , & Massot, M. (1998). Conspecific reproductive success and breeding habitat selection: Implications for the study of coloniality. Ecology, 79, 2415–2428.

[ece33241-bib-0031] Danchin, E. , & Cam, E. (2002). Can non‐breeding be a cost of breeding dispersal? Behavioral Ecology and Sociobiology, 51, 153–163.

[ece33241-bib-0032] Danchin, E. , Giraldeau, L. A. , Valone, T. J. , & Wagner, R. H. (2004). Public information: From nosy neighbors to cultural evolution. Science, 305, 487–491. 15273386 10.1126/science.1098254

[ece33241-bib-0033] Danchin, E. , Heg, D. , & Doligez, B. (2001). Public information and breeding habitat selection In Dispersal (Ed. Clobert J., Danchin E., Dhondt A. A. and Nichols J. D., pp. 243–258). Oxford University Press: Oxford.

[ece33241-bib-0034] Dittmann, T. , Zinsmeister, D. , & Becker, P. H. (2005). Dispersal decisions: Common terns, *Sterna hirundo*, choose between colonies during prospecting. Animal Behavior, 70, 13–20.

[ece33241-bib-0035] Doligez, B. , Cadet, C. , Danchin, E. , & Boulinier, T. (2003). When to use public information for breeding habitat selection? The role of environmental predictability and density dependence. Animal Behavior, 66, 973–988.

[ece33241-bib-0036] Doligez, B. , Danchin, E. , & Clobert, J. (2002). Public information and breeding habitat selection in a wild bird population. Science, 297, 1168–1170. 12183627 10.1126/science.1072838

[ece33241-bib-0037] Doligez, B. , Danchin, E. , Clobert, J. , & Gustafsson, L. (1999). The use of conspecific reproductive success for breeding habitat selection in a non‐colonial, hole‐nesting species, the collared flycatcher. Journal of Animal Ecology, 68, 1193–1206.

[ece33241-bib-0038] Doligez, B. , Pärt, T. , & Danchin, E. (2004). Prospecting in the collared flycatcher: Gathering public information for future breeding habitat selection? Animal Behavior, 67, 457–466.

[ece33241-bib-0039] Doligez, B. , Pärt, T. , Danchin, E. , Clobert, J. , & Gustafsson, L. (2004). Availability and use of public information and conspecific density for settlement decisions in the collared flycatcher. Journal of Animal Ecology, 73, 75–87.

[ece33241-bib-0040] Dunn, P. O. , & Hannon, S. J. (1991). Intraspecific competition and the maintenance of monogamy in tree swallows. Behavioral Ecology, 2, 258–266.

[ece33241-bib-0041] Dunn, P. O. , Robertson, R. J. , Michaud‐Freeman, D. , & Boag, P. T. (1994). Extra‐pair paternity in tree swallows: Why do females mate with more than one male? Behavioral Ecology and Sociobiology, 35, 273–281.

[ece33241-bib-0042] Dunn, P. O. , & Whittingham, L. A. (2005). Radio‐tracking of female tree swallows prior to egg‐laying. Journal of Field Ornithology, 76, 259–263.

[ece33241-bib-0043] Ghilain, A. , & Bélisle, M. (2008). Breeding success of tree swallows along a gradient of agricultural intensification. Ecological Applications, 18, 1140–1154. 18686577 10.1890/07-1107.1

[ece33241-bib-0044] Gimenez, O. , Viallefont, A. , Charmantier, A. , Pradel, R. , Cam, E. , Brown, C. R. , Anderson, M. D. , et al. (2008). The risk of flawed inference in evolutionary studies when detectability is less than one. American Naturalist, 172, 441–448. 10.1086/589520 18657010

[ece33241-bib-0045] Greenwood, P. J. , & Harvey, P. H. (1982). The natal and breeding dispersal of birds. Annual Review of Ecology and Systematics, 13, 1–21.

[ece33241-bib-0046] Halekoh, U. , Højsgaard, S. , & Yan, J. (2006). The R package geepack for generalized estimating equations. Journal of Statistical Software, 15, 1–11.

[ece33241-bib-0047] Hestbeck, J. B. , Nichols, J. D. , & Malecki, R. A. (1991). Estimates of movement and site fidelity using mark‐resight data of wintering Canada geese. Ecology, 72, 523–533.

[ece33241-bib-0048] Hoover, J. P. (2003). Decision rules for site fidelity in a migratory bird, the prothonotary warbler. Ecology, 84, 416–430.

[ece33241-bib-0049] Hussell, D. J. (1983). Age and plumage color in female tree swallows. Journal of Field Ornithology, 54, 312–318.

[ece33241-bib-0050] Johnson, M. L. , & Gaines, M. S. (1990). Evolution of dispersal: Theoretical models and empirical tests using birds and mammals. Annual Review of Ecology and Systematics, 21, 449–480.

[ece33241-bib-0051] Kempenaers, B. , Everding, S. , Bishop, C. , Boag, P. , & Robertson, R. J. (2001). Extra‐pair paternity and the reproductive role of male floaters in the tree swallow (*Tachycineta bicolor*). Behavioral Ecology and Sociobiology, 49, 251–259.

[ece33241-bib-0052] Kivelä, S. M. , Seppännen, J. T. , Ovaskainen, O. , Doligez, B. , Gustafsson, L. , Mönkkönen, M. , & Forsman, J. T. (2014). The past and the present in decision‐making: The use of conspecific and heterospecific cues in nest site selection. Ecology, 95, 3428–3439.

[ece33241-bib-0053] Lagrange, P. , Pradel, R. , Bélisle, M. , & Gimenez, O. (2014). Estimating dispersal among numerous sites using capture‐recapture data. Ecology, 95, 2316–2323. 25230481 10.1890/13-1564.1

[ece33241-bib-0054] Lecomte, N. , Gauthier, G. , & Giroux, J. F. (2008). Breeding dispersal in a heterogeneous landscape: The influence of habitat and nesting success in greater snow geese. Oecologia, 155, 33–41. 17938972 10.1007/s00442-007-0860-6

[ece33241-bib-0055] Leimar, O. , & Norberg, U. (1997). Metapopulation extinction and genetic variation in dispersal‐related traits. Oikos, 80, 448–458.

[ece33241-bib-0056] Lessard, A. , Bourret, A. , Bélisle, M. , Pelletier, F. , & Garant, D. (2014). Individual and environmental determinants of reproductive success in male tree swallow (*Tachycineta bicolor*). Behavioral Ecology and Sociobiology, 68, 733–742.

[ece33241-bib-0057] Matthysen, E. (2005). Density‐dependent dispersal in birds and mammals. Ecography, 28, 403–416.

[ece33241-bib-0058] McCarty, J. P. , & Winkler, D. W. (1999). Foraging ecology and diet selectivity of tree swallows feeding nestlings. Condor, 101, 246–254.

[ece33241-bib-0059] Montalvo, S. , & Potti, J. (1992). Breeding dispersal in Spanish Pied Flycatchers Ficedula hypoleuca. Ornis Scand, 23, 491–498.

[ece33241-bib-0060] Muldal, A. , Gibbs, H. L. , & Robertson, R. J. (1985). Preferred nest spacing of an obligate cavity‐nesting bird, the tree swallow. Condor, 87, 356–363.

[ece33241-bib-0061] Naves, L. C. , Monnat, J. Y. , & Cam, E. (2006). Breeding performance, mate fidelity, and nest site fidelity in a long‐lived seabird: Behaving against the current? Oikos, 115, 263–276.

[ece33241-bib-0062] Nocera, J. J. , Forbes, G. J. , & Giraldeau, L.‐A. (2006). Inadvertent social information in breeding site selection of natal dispersing birds. Proceedings of the Royal Society of London. Series B, Biological Sciences, 273, 349–355. 16543178 10.1098/rspb.2005.3318 PMC1560037

[ece33241-bib-0063] Nocera, J. J. , Forbes, G. J. , & Giraldeau, L.‐A. (2009). Aggregations from using inadvertent social information: A form of ideal habitat selection. Ecography, 32, 143–152.

[ece33241-bib-0064] Paradis, E. , Baillie, S. , Sutherland, W. , & Gregory, R. (1998). Patterns of natal and breeding dispersal in birds. Journal of Animal Ecology, 67, 518–536.

[ece33241-bib-0065] Parejo, D. , White, J. , Clobert, J. , Dreiss, A. , & Danchin, E. (2007). Blue tits use fledgling quantity and quality as public information in breeding site choice. Ecology, 88, 2373–2382. 17918414 10.1890/06-2000.1

[ece33241-bib-0066] Pärt, T. , Arlt, D. , Doligez, B. , Low, M. , & Qvarnström, A. (2011). Prospectors combine social and environmental information to improve habitat selection and breeding success in the subsequent year. Journal of Animal Ecology, 80, 1227–1235. 21569028 10.1111/j.1365-2656.2011.01854.x

[ece33241-bib-0067] Pärt, T. , & Doligez, B. (2003). Gathering public information for habitat selection: Prospecting birds cue on parental activity. Proceedings of the Royal Society of London. Series B, Biological Sciences, 270, 1809–1813. 12964983 10.1098/rspb.2003.2419 PMC1691451

[ece33241-bib-0068] Pärt, T. , & Gustafsson, L. (1989). Breeding dispersal in the collared flycatcher (*Ficedula albicollis*): Possible causes and reproductive consequences. Journal of Animal Ecology, 58, 305–320.

[ece33241-bib-0069] Ponchon, A. , Grémillet, D. , Doligez, B. , Chambert, T. , Tveraa, T. , González‐Solís, J. , & Boulinier, T. (2013). Tracking prospecting movements involved in breeding habitat selection: Insights, pitfalls and perspectives. Methods in Ecology and Evolution, 4, 143–150.

[ece33241-bib-0070] Pradel, R. (2005). Multievent: An extension of multistate capture‐recapture models to uncertain states. Biometrics, 61, 442–447. 16011690 10.1111/j.1541-0420.2005.00318.x

[ece33241-bib-0071] Pradel, R. , Gimenez, O. , & Lebreton, J. (2005). Principles and interest of GOF tests for multistate capture‐recapture models. Animal Biodiversity and Conservation, 28, 189–204.

[ece33241-bib-1000] R Core Team (2013). R: A language and environment for statistical computing. R Foundation for Statistical Computing, Vienna, Austria ISBN 3‐900051‐07‐0, URL http://www.R-project.org.

[ece33241-bib-0072] Racine, F. , Giraldeau, L.‐A. , Patenaude‐Monette, M. , & Giroux, J.‐F. (2012). Evidence of social information on food location in a ring‐billed gull colony, but the birds do not use it. Animal Behavior, 84, 175–182.

[ece33241-bib-0073] Rieucau, G. , & Giraldeau, L.‐A. (2011). Exploring the costs and benefits of social information use: An appraisal of current experimental evidence. Philosophical transactions of the Royal Society of London. Series B, Biological Sciences, 1567, 949–957. 10.1098/rstb.2010.0325 PMC3049093 21357217

[ece33241-bib-0074] Robertson, R. J. , Gibbs, H. L. , & Stutchbury, B. J. (1986). Spitefulness, altruism, and the cost of aggression: Evidence against superterritoriality in tree swallows. Condor, 88, 104–105.

[ece33241-bib-0075] Robertson, R. J. , & Rendell, W. B. (1990). A comparison of the breeding ecology of a secondary cavity nesting bird, the tree swallow (*Tachycineta bicolor*), in nest boxes and natural cavities. Canadian Journal of Zoology, 68, 1046–1052.

[ece33241-bib-0076] Robillard, A. , Garant, D. , & Bélisle, M. (2013). The swallow and the sparrow: How agricultural intensification affects abundance, nest site selection and competitive interactions. Landscape Ecology, 28, 201–215.

[ece33241-bib-0077] Sanz‐Aguilar, A. , Tavecchia, G. , Genovart, M. , Igual, J. M. , Oro, D. , Rouan, L. , & Pradel, R. (2011). Studying the reproductive skipping behavior in long‐lived birds by adding nest inspection to individual‐based data. Ecological Applications, 21, 555–564. 21563585 10.1890/09-2339.1

[ece33241-bib-0078] Schaub, M. , & Von Hirschheydt, J. (2009). Effect of current reproduction on apparent survival, breeding dispersal, and future reproduction in barn swallows assessed by multistate capture–recapture models. Journal of Animal Ecology, 78, 625–635. 19040683 10.1111/j.1365-2656.2008.01508.x

[ece33241-bib-0079] Schjørring, S. , Gregersen, J. , & Bregnballe, T. (1999). Prospecting enhances breeding success of first‐time breeders in the great cormorant, *Phalacrocorax carbo sinensis* . Animal Behavior, 57, 647–654. 10.1006/anbe.1998.0993 10196055

[ece33241-bib-0080] Serrano, D. , Tella, J. L. , Forero, M. G. , & Donazar, J. A. (2001). Factors affecting breeding dispersal in the facultatively colonial lesser kestrel: Individual experience vs. conspecific cues. Journal of Animal Ecology, 70, 568–578.

[ece33241-bib-0081] Shutler, D. , & Clark, R. G. (2003). Causes and consequences of tree swallow (*Tachycineta bicolor*) dispersal in Saskatchewan. Auk, 120, 619–631.

[ece33241-bib-0082] Stamps, J. A. (1988). Conspecific attraction and aggregation in territorial species. American Naturalist, 131, 329–347.

[ece33241-bib-0083] Stamps, J. A. , Krishnan, V. V. , & Reid, M. L. (2005). Search costs and habitat selection by dispersers. Ecology, 86, 510–518.

[ece33241-bib-0084] Steven, D. (1978). The influence of age on the breeding biology of the tree swallow *Iridoprocne bicolor* . Ibis, 120, 516–523.

[ece33241-bib-0085] Stutchbury, B. J. , & Robertson, R. J. (1987). Behavioral tactics of subadult female floaters in the tree swallow. Behavioral Ecology and Sociobiology, 20, 413–419.

[ece33241-bib-0086] Switzer, P. V. (1993). Site fidelity in predictable and unpredictable habitats. Evolutionary Ecology, 7, 533–555.

[ece33241-bib-0087] Switzer, P. V. (1997). Past reproductive success affects future habitat selection. Behavioral Ecology and Sociobiology, 40, 307–312.

[ece33241-bib-0088] Wagner, R. H. , & Danchin, E. (2010). A taxonomy of biological information. Oikos, 119, 203–209.

[ece33241-bib-0089] Ward, M. P. (2005). Habitat selection by dispersing yellow‐headed blackbirds: Evidence of prospecting and the use of public information. Oecologia, 145, 650–657. 16007408 10.1007/s00442-005-0179-0

[ece33241-bib-0090] Winkler, D. W. , Hallinger, K. K. , Ardia, D. R. , Robertson, R. J. , Stutchbury, B. J. , & Cohen, R. R. (2011). Tree swallow (Tachycineta bicolor) in The Birds of North America Online. Ithaca, NY: Cornell Lab of Ornithology.

[ece33241-bib-0091] Winkler, D. W. , Wrege, P. H. , Allen, P. E. , Kast, T. L. , Senesac, P. , Wasson, M. F. , Llambías, P. E. , et al. (2004). Breeding dispersal and philopatry in the tree swallow. Condor, 106, 768–776.

[ece33241-bib-0092] Yoder, J. M. , Marschall, E. A. , & Swanson, D. A. (2004). The cost of dispersal: Predation as a function of movement and site familiarity in ruffed grouse. Behavioral Ecology, 15, 469–476.

[ece33241-bib-0093] Zollner, P. A. , & Lima, S. L. (2005). Behavioral tradeoffs when dispersing across a patchy landscape. Oikos, 108, 219–230.

