# Supplementary material for: Assessment of individual and conspecific reproductive success as determinants of breeding dispersal of female tree swallows: A capture–recapture approach
Source: Ecol Evol. 2017 Aug 9;7(18):7334–46. doi: 10.1002/ece3.3241 (PMC5606858; doi:10.1002/ece3.3241)
Supplement: Supplementary file 1 [file ECE3-7-7334-s001.docx]

Supplementary material

For : Assessment of individual and conspecific reproductive success as determinants of breeding dispersal of female tree swallows: a capture-recapture approach

By : Paméla Lagrange, Olivier Gimenez, Blandine Doligez, Roger Pradel, Dany Garant, Fanie Pelletier, Marc Bélisle

### Appendix S1: Description of the RSi and RSc in our study system in southern Québec, Canada.


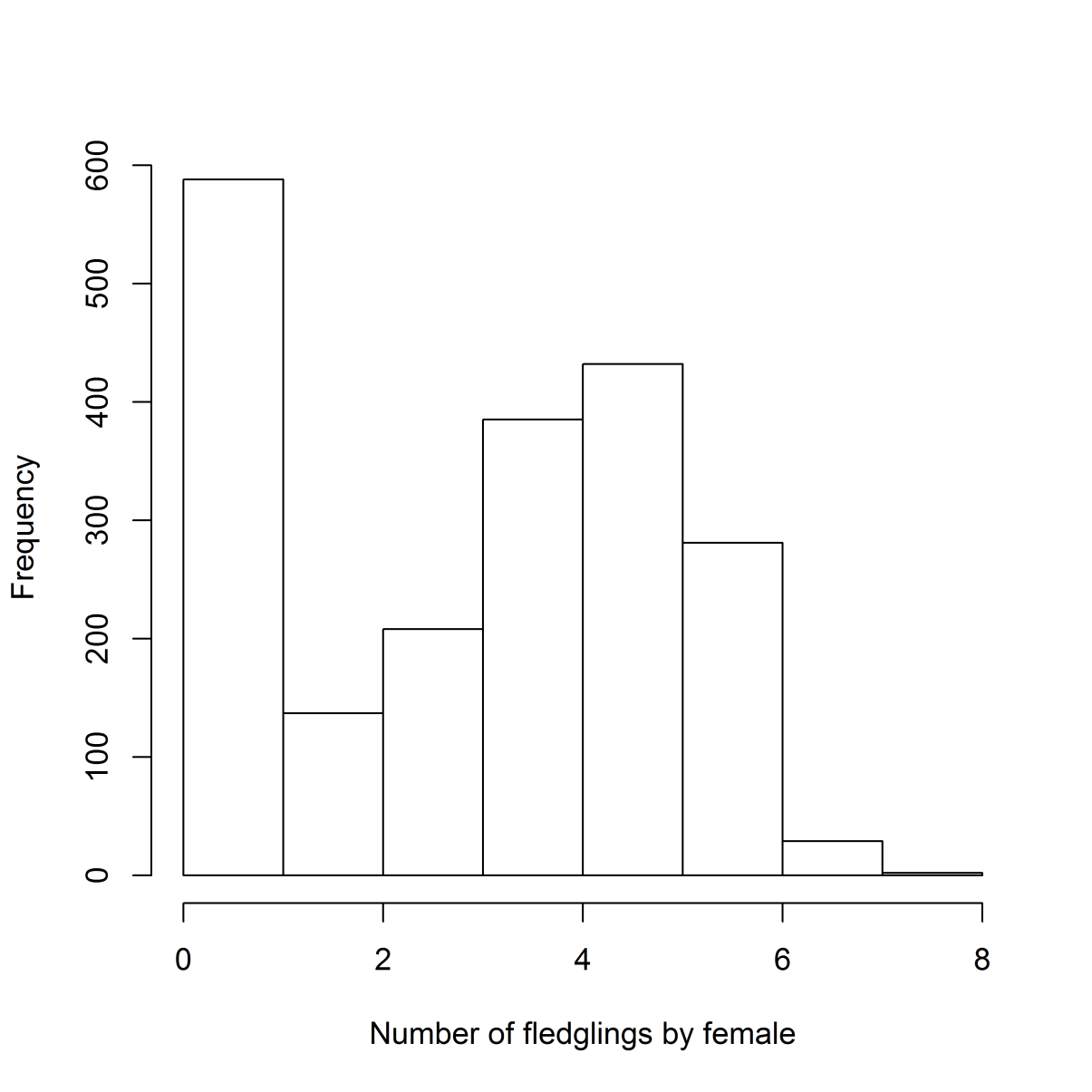


**Figure S1: Distribution of the annual number of fledglings (RSi) produced by female tree swallows breeding in southern Québec, Canada, between 2004 and 2013.**


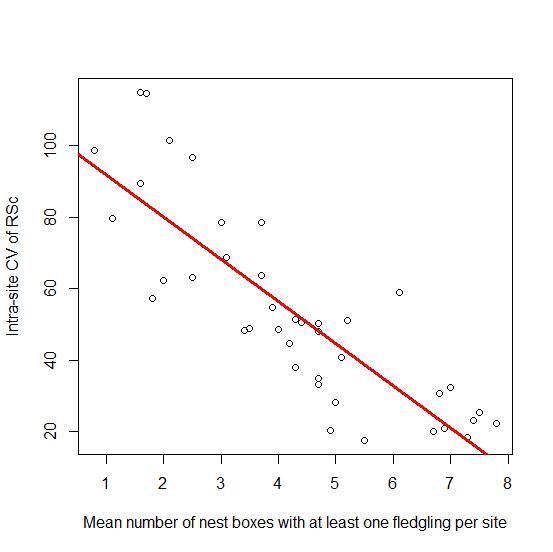


**Figure S2: Between-year variability of the conspecific reproductive success (RSc) of each site (farm) as a function of the mean number of nest boxes with at least one fledgling per site where female tree swallows bred between 2004 and 2013 in southern Québec, Canada.** Each point represents the coefficient of variation (%) of the mean RSc of a given site over ten years as a function of its annual mean number of nest boxes that produced at least one fledgling. The line depicts the predictions of a simple linear regression.

### Appendix S2: Capture-Recapture method


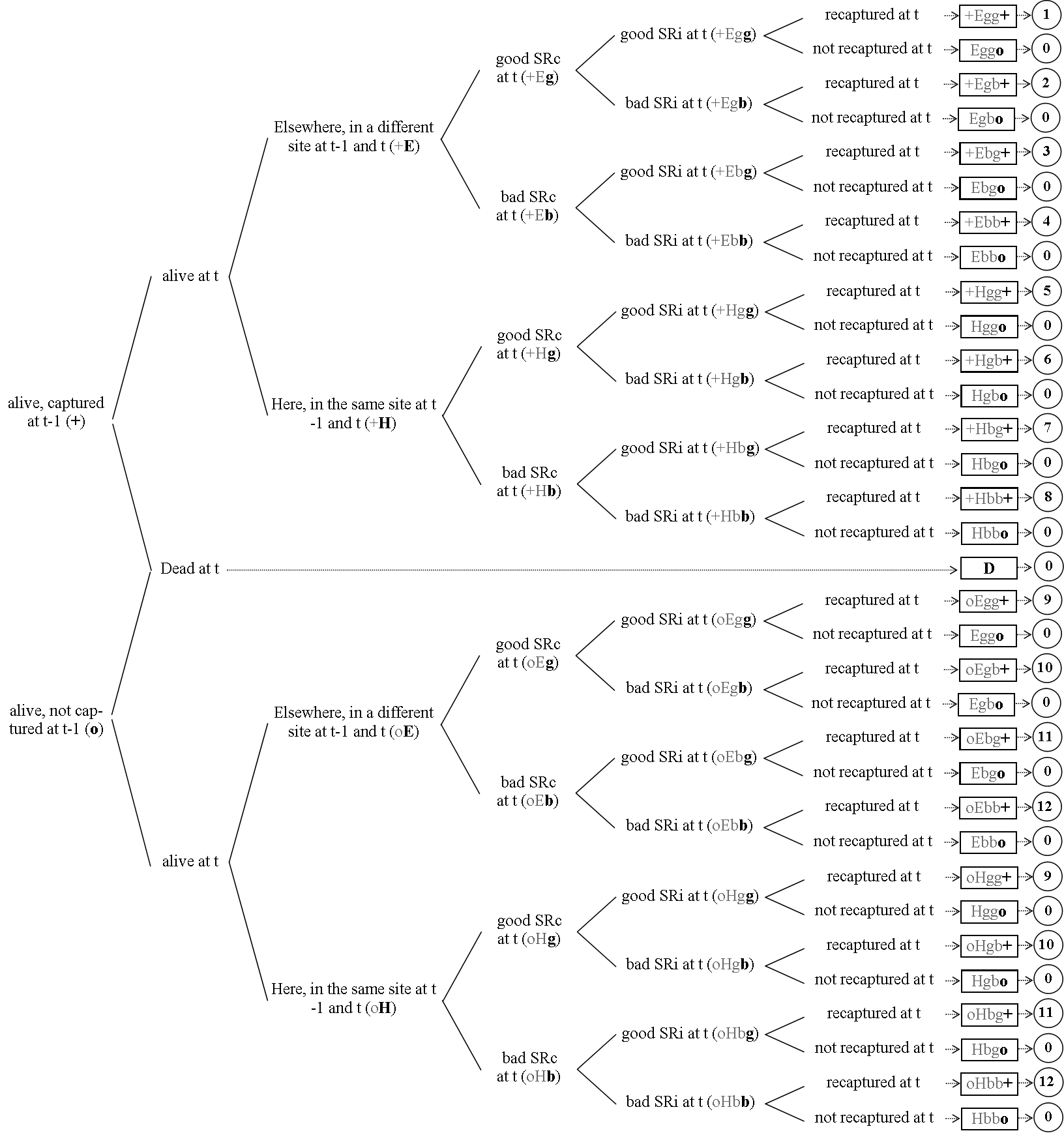


**Figure S3: Transition steps of an individual from t - 1 to t and explanation of states associated with events.** The diagram shows the steps leading to the observation (at t - 1 and t) of an individual: survival (alive or dead), fidelity (Here or Elsewhere), RSc (good or bad), RSi (good or bad) and recapture (recaptured or not). Each updated information appears in bold while the old one is grayed out. We end up in the last step with 25 states (in boxes) that can generate 13 events (numbered circles). We denoted the composite states by prefixing the previous capture status (detected: ‘+’ or not detected: ‘o’ at t - 1), secondly by the dispersal status (Here: ‘H’, occupying the same site or Elsewhere: ‘E’, occupying a different site), thirdly by the RSc (if the site showed a bad RSc at t: ‘b’ or a good one: ‘g’), then by the RSi (if the individual had a bad reproduction at t: ‘b’ or a good one: ‘g’) and lastly by the current capture status (if detected: ‘+’ or if not detected: ‘o’ at t).

**Figure S4: Matrices implemented in E-SURGE to estimate the probability to be in each state initially (π), to survive at t (S), to move or not between t and t + 1 (F), to keep or not the same RSi (I) and RSc (C) between t and t + 1, to be captured (R) at t + 1 and to be detected in the last step to link events and states.** All unknown information appears in grey and for each step (or new matrix), the information is actualized in black bold. Known and irrelevant information for a given matrix appear in black.

### Appendix S3: Model selection in Capture-Recapture analyses

**Table S1: Forward model selection of the Capture-Recapture analysis used to determine the basic structure of demographic parameters of female tree swallows breeding in southern Québec, Canada, 2004-2013**. Each model is defined by five parameters: S, F, I, C and R. The effects were tested alone, in addition (+) or in interaction (*). A constant parameter is represented by ‘i’. k is the number of parameters of each model and ΔQAIC_c_ (ĉ = 1.94) gives the difference between the QAIC_c_ value of the model and that of the model with the lowest QAIC_c_ value (i.e., Model_55_, QAIC_c_ = 4265.80). # is the model number. The best model for each parameter is in bold and the null model is #11. The best model structure for a given parameter was retained before moving to the next parameters.

| Parameters | Effects | k | ΔQAIC_c_ | *#* |
| --- | --- | --- | --- | --- |
| Recapture (R) | i | 11 | 176.96 | 11 |
|  | **RSi** | **12** | **124.22** | **14** |
|  | t | 19 | 191.67 | 17 |
|  | RSi+t | 20 | 128.90 | 18 |
|  | RSi*t | 28 | 140.82 | 19 |
| Transition between a good/bad RSc (C) | **RSc** | **13** | **81.96** | **20** |
|  | t | 20 | 135.78 | 21 |
|  | RSc+t | 21 | 94.22 | 22 |
|  | RSc*t | 29 | 105.27 | 23 |
| Transition between a good/bad RSi (I) | RSi | 14 | 83.91 | 24 |
|  | RSc | 14 | 80.14 | 25 |
|  | memory | 14 | 79.69 | 26 |
|  | **RSi*RSc** | **16** | **67.80** | **27** |
|  | RSc*memory | 16 | 74.77 | 28 |
|  | RSi*memory | 16 | 82.98 | 29 |
|  | RSi*RSc*memory | 20 | 68.57 | 30 |
|  | t | 21 | 90.19 | 31 |
|  | age | 14 | 83.99 | 32 |
|  | RSi*RSc+t | 24 | 76.99 | 33 |
|  | RSi*RSc*t | 48 | 100.95 | 34 |
|  | RSi*RSc*age | 20 | 72.94 | 35 |
| Survival (S) | memory | 19 | 66.35 | 38 |
|  | **RSi** | **17** | **25.17** | **39** |
|  | RSi*memory | 21 | 30.52 | 44 |
|  | t | 24 | 68.19 | 45 |
|  | age | 17 | 67.20 | 46 |
|  | RSi+t | 25 | 30.86 | 47 |
|  | RSi*t | 33 | 42.07 | 48 |
|  | RSi*age | 19 | 27.05 | 49 |
| Fidelity (F) | memory | 20 | 10.94 | 52 |
|  | RSi | 18 | 7.46 | 53 |
|  | RSc | 18 | 22.19 | 54 |
|  | **RSi*memory** | **22** | **0** | **55** |
|  | RSc*memory | 22 | 11.859 | 56 |
|  | RSi*RSc | 20 | 9.0016 | 57 |
|  | RSi*RSc*memory | 26 | 7.2234 | 58 |
|  | t | 25 | 30.393 | 59 |
|  | age | 18 | 27.001 | 60 |
|  | RSi*memory+t | 30 | 3.0204 | 61 |
|  | RSi*memory*t | 52 | 36.487 | 62 |
|  | RSi*memory*age | 24 | 2.4797 | 63 |

**Table S2: Second forward model selection of the Capture-Recapture analysis used to evaluate the effects of previous dispersal behavior, age (SY: second year** vs. **ASY: after second year) and individual reproductive success (RSi) on apparent survival (S) and transition between a good/bad RSi (I) of female tree swallows breeding in southern Québec, Canada, between 2004-2013.**

Model structures for R, C and F were obtained from the first model selection (i.e., as in Model #55, Table S1). We retested the effect of the variables alone, in addition (+) or in interaction (*) on S and I. A constant parameter is represented by ‘i’. k is the number of parameters of each model used to calculate the ΔQAIC_c_ (ĉ = 1.94) giving the difference between the QAIC_c_ value of the model and that of the model with the lowest QAIC (i.e., Model_55_, QAIC_c_ = 4265.80). # is the model number. The best model for each parameter is in bold.

| Parameters | Effects | k | Δ QAIC_c_ | *#* |
| --- | --- | --- | --- | --- |
| Survival (S) | i | 21 | 42.66 | S11 |
|  | memory | 22 | 36.88 | S38 |
|  | **RSi** | **22** | **0.00** | **55** |
|  | RSi*memory | 24 | 0.91 | S44 |
|  | t | 29 | 43.16 | S12 |
|  | age | 22 | 42.08 | S13 |
|  | RSi+t | 30 | 5.84 | S47 |
|  | RSi*t | 38 | 17.17 | S48 |
|  | (RSi)*age | 24 | 1.95 | S49 |
| Transition between a good/bad RSi (I) | i | 19 | 14.55 | I20 |
|  | RSi | 20 | 16.59 | I24 |
|  | RSc | 20 | 12.22 | I25 |
|  | memory | 20 | 16.51 | I26 |
|  | **RSi*RSc** | **22** | **0.00** | **55** |
|  | RSc*memory | 22 | 9.24 | I28 |
|  | RSi*memory | 22 | 19.32 | I29 |
|  | RSi*RSc*memory | 26 | 3.83 | I30 |
|  | t | 27 | 25.02 | I31 |
|  | Age | 20 | 16.42 | I32 |
|  | RSi*RSc+t | 34 | 11.93 | I33 |
|  | RSi*RSc*t | 90 | 81.27 | I34 |
|  | RSi*RSc*age | 34 | 17.09 | I35 |

**Table S3: Forward model selection of the Capture-Recapture analysis used to determine the basic structure of demographic parameters of female tree swallows breeding in southern Québec, Canada, 2004-2013, with RSc standardized by the number of occupied nestboxes**. Operationally, RSc was defined as good if the number of nestboxes with at least one fledgling of a site (farm) divided by the density of occupied nestboxes on that site was greater or equal to the annual median of this ratio across all sites. Each model is defined by five parameters: S, F, I, C and R. The effects of RSi, RSc, previous dispersal behavior (memory) and age were tested alone, in addition (+) or in interaction (*) as in the table S1. A constant parameter is represented by ‘i’. k is the number of model parameters used to calculate the ΔQAIC_c_ (ĉ = 1.94; Model55 showed the lowest QAIC_c_ = 4491.71) and corresponding Akaike weight (w_i_) of each model. # is the model number. The best model for each parameter is in bold and the null model is #11.

| Parameters | Effects | k | Δ QAIC_c_ | w_i_ | *#* |
| --- | --- | --- | --- | --- | --- |
| Recapture (R) | i | 11 | 152.69 | 0.00 | 11 |
|  | **RSi** | **12** | **99.94** | **0.00** | **14** |
|  | t | 19 | 167.39 | 0.00 | 17 |
|  | RSi+t | 20 | 104.63 | 0.00 | 18 |
|  | RSi*t | 28 | 116.55 | 0.00 | 19 |
| Transition between a good/bad RSc (C) | i | 12 | 99.94 | 0.00 | 14 |
|  | **RSc** | **13** | **85.97** | **0.00** | **20** |
|  | t | 20 | 101.82 | 0.00 | 21 |
|  | RSc+t | 21 | 90.25 | 0.00 | 22 |
|  | RSc*t | 29 | 88.70 | 0.00 | 23 |
| Transition between a good/bad RSi (I) | i | 13 | 85.97 | 0.00 | 20 |
|  | RSi | 14 | 87.91 | 0.00 | 24 |
|  | RSc | 14 | 82.99 | 0.00 | 25 |
|  | memory | 14 | 83.69 | 0.00 | 26 |
|  | **RSi*RSc** | **16** | **69.44** | **0.00** | **27** |
|  | RSc*memory | 16 | 78.30 | 0.00 | 28 |
|  | RSi*memory | 16 | 86.98 | 0.00 | 29 |
|  | RSi*RSc*memory | 20 | 71.60 | 0.00 | 30 |
|  | t | 21 | 94.19 | 0.00 | 31 |
|  | age | 14 | 87.99 | 0.00 | 32 |
|  | RSi*RSc+t | 24 | 76.74 | 0.00 | 33 |
|  | RSi*RSc*t | 48 | 102.13 | 0.00 | 34 |
|  | RSi*RSc*age | 20 | 73.59 | 0.00 | 35 |
| Survival (S) | i | 16 | 69.44 | 0.00 | 27 |
|  | memory | 19 | 60.35 | 0.00 | 38 |
|  | **RSi** | **17** | **26.11** | **0.00** | **39** |
|  | RSi*memory | 21 | 29.99 | 0.00 | 44 |
|  | t | 24 | 69.62 | 0.00 | 45 |
|  | age | 17 | 68.67 | 0.00 | 46 |
|  | RSi+t | 25 | 31.61 | 0.00 | 47 |
|  | RSi*t | 33 | 42.84 | 0.00 | 48 |
|  | RSi*age | 19 | 27.85 | 0.00 | 49 |
| Fidelity (F) | i | 17 | 26.11 | 0.00 | 39 |
|  | memory | 20 | 9.01 | 0.01 | 52 |
|  | RSi | 18 | 8.40 | 0.01 | 53 |
|  | RSc | 18 | 21.78 | 0.00 | 54 |
|  | **RSi*memory** | **22** | **0.00** | **0.66** | **55** |
|  | RSc*memory | 22 | 8.55 | 0.01 | 56 |
|  | RSi*RSc | 20 | 9.27 | 0.01 | 57 |
|  | RSi*RSc*memory | 26 | 5.18 | 0.05 | 58 |
|  | t | 25 | 31.33 | 0.00 | 59 |
|  | age | 18 | 27.94 | 0.00 | 60 |
|  | RSi*memory+t | 30 | 3.55 | 0.11 | 61 |
|  | RSi*memory*t | 53 | 40.16 | 0.00 | 62 |
|  | RSi*memory*age | 24 | 3.16 | 0.14 | 63 |

### Appendix S4: Standard approach assuming perfect detection

**Methods**

We assessed the influence of assuming a perfect detectability of marked female tree swallows breeding in southern Québec, Canada, between 2004-2013 when estimating dispersal probabilities and the effects of ecological variables on the latter by comparing the results of Capture-Recapture analyses with those obtained by generalized linear mixed models (i.e., GLMMs with logit link function and binomial error structure; see Gimenez et al. (2008) for a similar exercise). GLMMs modeled whether females dispersed or not as a function of female individual reproductive success (RSi; good or bad), conspecific reproductive success (RSc; good or bad), female age (SY vs. ASY), memory (disperser or faithful at t - 1), and the interactions between RSi and memory as well as between RSi and RSc. Year of capture was considered a random effect. We also used two datasets to assess the sensitivity of GLMM results with respect to case selection. The first dataset (Dataset #1) was limited to 632 individuals captured two consecutive years and included 14 % of dispersers based on 632 events of recapture. In this dataset, dispersal cannot be confounded with lack of survival but individuals not recaptured at t + 1 because they dispersed outside the study area or simply went undetected can still bias (likely underestimate) dispersal probabilities. The second dataset (Dataset #2) included individuals captured at least twice but not necessarily in two consecutive years. With this dataset, we could assume that individuals not recaptured two consecutive years were dispersers. The dataset thereby included 667 individuals of which 23 % were dispersers. This dataset has the advantage over the former of potentially alleviating the bias resulting from birds dispersing outside the study area but can also lead to overestimated dispersal probabilities via individuals that went undetected but did not disperse. Model selection was based on a list of models that included all combinations of explanatory variables as main effects and two-way interactions. As for multievent analyses, we used multimodel inference based on AIC_c_ to estimate model-averaged regression coefficients using package MuMIn 1.9.13 in R 3.0.2 (R Development Core Team 2013).

**Table S4: Model selection of GLMMs estimating the probability of dispersal of female tree swallows breeding in southern Québec, Canada, as a function of individual reproductive success (RSi), conspecific reproductive success (RSc), previous dispersal behavior (memory; faithful or not) and age (SY: second year** vs. **ASY: after second year) for two datasets.** All models included year of capture as random effect. # is the model name of the model. Dataset #1 included individuals captured two consecutive years, and Dataset #2, individuals captured at least two times but not necessarily in two consecutive years. The number of parameters (k) and deviance were used to calculate the AIC_c_ and Akaike weight (w_i_) of each model. The model showing the lowest AIC_c_ was m13 for both datasets (Dataset #1: AIC_c_ = 447.0; Dataset #2: AIC_c_ = 579.40).

| Models | # | k | Dataset #1 (N=632) | | Dataset #2 (N=667) | |
| --- | --- | --- | --- | --- | --- | --- |
|  |  |  | Δ AIC_c_ | w_i_ | Δ AIC_c_ | w_i_ |
| RSi+RSc+memory | m13 | 6 | 0.00 | 0.25 | 0.00 | 0.20 |
| RSi+RSc+memory+RSi*RSc | m3 | 7 | 0.39 | 0.20 | 0.34 | 0.17 |
| RSi+memory | m18 | 5 | 0.80 | 0.16 | 0.86 | 0.13 |
| RSi+RSc+memory+age | m12 | 7 | 1.91 | 0.09 | 2.04 | 0.07 |
| RSi+RSc+memory+age+RSi*RSc | m2 | 8 | 2.25 | 0.08 | 2.39 | 0.06 |
| RSi+memory+age | m15 | 6 | 2.75 | 0.06 | 2.89 | 0.05 |
| RSi+RSc+memory+RSi*RSc+RSi*memory | m11 | 9 | 3.85 | 0.04 | 1.53 | 0.09 |
| RSi+RSc+memory+RSi*memory | m7 | 8 | 3.42 | 0.04 | 1.38 | 0.10 |
| RSi+memory+RSi*memory | m9 | 7 | 4.13 | 0.03 | 2.45 | 0.06 |
| RSi+RSc+memory+age+RSi*memory | m6 | 9 | 5.34 | 0.02 | 3.43 | 0.04 |
| RSi+RSc+memory+age+RSi*RSc+RSi*memory | m10 | 10 | 5.72 | 0.01 | 3.58 | 0.03 |
| RSi+memory+age+RSi*memory | m8 | 8 | 6.08 | 0.01 | 4.50 | 0.02 |
| null | m1 | 1 | 65.10 | 0.00 | 60.22 | 0.00 |
| RSi+RSc+age | m14 | 5 | 26.63 | 0.00 | 17.24 | 0.00 |
| RSc+memory+age | m16 | 6 | 31.18 | 0.00 | 37.64 | 0.00 |
| RSi+RSc | m17 | 4 | 25.10 | 0.00 | 16.07 | 0.00 |
| RSi+age | m19 | 4 | 28.60 | 0.00 | 19.35 | 0.00 |
| RSc+memory | m20 | 5 | 29.22 | 0.00 | 35.63 | 0.00 |
| RSc+age | m21 | 4 | 56.42 | 0.00 | 54.17 | 0.00 |
| memory+age | m22 | 5 | 35.60 | 0.00 | 43.22 | 0.00 |
| RSi | m23 | 3 | 27.22 | 0.00 | 18.39 | 0.00 |
| RSc | m24 | 3 | 55.00 | 0.00 | 53.28 | 0.00 |
| memory | m25 | 4 | 33.63 | 0.00 | 41.23 | 0.00 |
| age | m26 | 3 | 63.45 | 0.00 | 62.62 | 0.00 |
| RSi+RSc+age+RSi*RSc | m4 | 6 | 27.89 | 0.00 | 18.41 | 0.00 |
| RSi+RSc+RSi*RSc | m5 | 5 | 26.32 | 0.00 | 17.15 | 0.00 |

**Table S5: Model-averaged parameters of the GLMM approach estimating the probability of dispersal of female tree swallows breeding in southern Québec, Canada, for (A) Dataset #1 (N = 632) and (B) Dataset #2 (N = 667).** Unconditional standard errors (SE) and confidence intervals are given for each explanatory variable and the intercept.

A)

| Variables | Estimates | SE | CI (-) | CI (+) |
| --- | --- | --- | --- | --- |
| (Intercept) | 0.52 | 0.75 | -0.95 | 1.99 |
| RSi | -1.84 | 0.68 | -3.18 | -0.51 |
| RSc | -0.76 | 0.53 | -1.80 | 0.28 |
| memory(faithful) | -2.05 | 0.79 | -3.59 | -0.51 |
| RSc*RSi | 0.84 | 0.66 | -0.45 | 2.14 |
| ageSY | -0.14 | 0.36 | -0.85 | 0.58 |
| memory(faithful)*RSi | -1.11 | 1.37 | -3.79 | 1.57 |

B)

| Variables | Estimates | SE | CI (-) | CI (+) |
| --- | --- | --- | --- | --- |
| (Intercept) | 0.44 | 0.82 | -1.17 | 2.05 |
| RSi | -1.51 | 0.86 | -3.20 | 0.17 |
| RSc | -0.69 | 0.48 | -1.63 | 0.25 |
| memory(faithful) | -1.35 | 0.79 | -2.90 | 0.20 |
| RSc*RSi | 0.76 | 0.58 | -0.38 | 1.90 |
| memory(faithful)*RSi | -0.30 | 1.26 | -2.79 | 2.18 |
| ageSY | 0.00 | 0.31 | -0.60 | 0.60 |


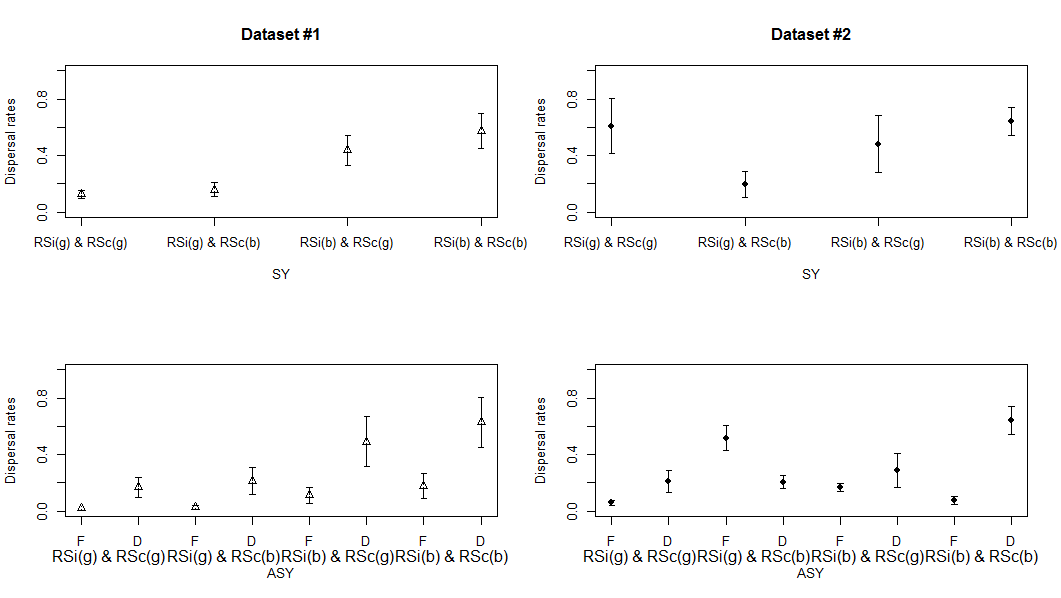


**Figure S5: Dispersal probabilities of female tree swallows breeding in southern Québec, Canada, between 2004 and 2012 according to good/bad individual reproductive success (RSi(g)/RSi(b)), good/bad conspecifics reproductive success (RSc(g)/RSc(b)), female age (SY: second year** vs. **ASY: after second year) and previous dispersal behavior (faithful or not)**. Estimations stem from multimodel inference applied to GLMMs fitted to Dataset #1 (females captured two consecutive years; empty triangles) and to Dataset #2 (females captured not necessarily in two consecutive years; black circles). For each combination of reproductive status, we indicate in abscissa the dispersal status in the previous reproduction (D if females dispersed the previous year, F, if females were faithful). The dispersal status is unknown for SY birds.
